# Cold acclimation alters DNA methylation patterns and confers tolerance to heat and increases growth rate in *Brassica rapa*

**DOI:** 10.1093/jxb/erw496

**Published:** 2017-02-01

**Authors:** Tongkun Liu, Ying Li, Weike Duan, Feiyi Huang, Xilin Hou

**Affiliations:** 1 Department of Horticulture, Nanjing Agricultural University, Nanjing, China

**Keywords:** *Brassica rapa*, cold acclimation, DNA methylation, heat tolerance, organic acids, photosynthesis.

## Abstract

Epigenetic modifications are implicated in plant adaptations to abiotic stresses. Exposure of plants to one stress can induce resistance to other stresses, a process termed cross-adaptation, which is not well understood. In this study, we aimed to unravel the epigenetic basis of elevated heat-tolerance in cold-acclimated *Brassica rapa* by conducting a genome-wide DNA methylation analysis of leaves from control (CK) and cold-acclimated (CA) plants. We found that both methylation and demethylation occurred during cold acclimation. Two significantly altered pathways, malate dehydrogenase activity and carbon fixation, and 1562 differentially methylated genes, including *BramMDH1*, *BraKAT2*, *BraSHM4*, and *Bra4CL2*, were identified in CA plants. Genetic validation and treatment of *B. rapa* with 5-aza-2-deoxycytidine (Aza) suggested that promoter demethylation of four candidate genes increased their transcriptional activities. Physiological analysis suggested that elevated heat-tolerance and high growth rate were closely related to increases in organic acids and photosynthesis, respectively. Functional analyses demonstrated that the candidate gene *BramMDH1* (mMDH: mitochondrial malate dehydrogenase) directly enhances organic acids and photosynthesis to increase heat-tolerance and growth rate in *Arabidopsis*. However, Aza-treated *B. rapa*, which also has elevated *BramMDH1* levels, did not exhibit enhanced heat-tolerance. We therefore suggest that DNA demethylation alone is not sufficient to increase heat-tolerance. This study demonstrates that altered DNA methylation contributes to cross-adaptation.

## Highlight

Changes in DNA methylation contribute to cross-adaptation in cold-acclimated *Brassica rapa*, which has elevated organic acids and increased photosynthesis, and confers tolerance to heat and enhances growth rate.

## Introduction

Exposure of plants to a moderate stress can induce resistance to other stresses, a phenomenon termed cross-adaptation. This is an aspect of environmental physiology that has not been explored extensively. Low temperature is a major environmental stress that seriously compromises plant development, distribution, and productivity. Many plants exhibit increased freezing tolerance upon exposure to low, non-freezing temperatures, a process known as cold acclimation. This is a complex process that involves many changes, ranging from changes in gene expression to those in physiological, biochemical, and metabolic processes (Chinnusamy *et al.*, 2007; Hua, 2009; Medina *et al.*, 2011; Knight and Knight, 2012; Shi *et al.*, 2015). These include changes in *SFR6* and *CBF* gene expression (Fowler and Thomashow, 2002; Knight *et al.*, 2009; Jia *et al.*, 2016; Zhao *et al.*, 2016), photosynthesis (Allen and Ort, 2001), and the stability of membranes and the cytoskeleton (Nishida and Murata, 1996; Thalhammer and Hincha, 2014), as well as metabolic adjustments including the production of hormones (Barrero-Gil *et al.*, 2016) and organic acids (Nagler *et al.*, 2015). During cold acclimation, the suppression of photosynthesis and photosynthetic gene expression is removed in *Arabidopsis* leaves (Strand *et al.*, 1997). Cold acclimation also increases cytoplasmic volume, accompanying increases in the activities of enzymes in the Calvin cycle and in the sucrose biosynthesis pathway (Strand *et al.*, 1999). In addition, elevated levels of organic acids (alpha-ketoglutarate, fumarate, malate, and citrate) have been detected in cold-acclimated *Arabidopsis* (Nagler *et al.*, 2015; Dyson *et al.*, 2016). However, the mechanisms by which plants sense low temperatures and subsequently adjust photosynthesis and metabolism remain to be determined.

Interestingly, after cold acclimation, plants can exhibit increased resistance not only to freezing but also to heat stress (Palta *et al.*, 1981; Fu *et al.*, 1998). For example, in winter rye, cold acclimation increases plant heat tolerance; this is not attributable to elevated heat-shock proteins, which are not induced by cold acclimation and therefore are not involved in the increased heat tolerance observed. However, a number of heat-stable proteins, sugars, and long-chain carbohydrate polymers accumulate during the cold acclimation process and may play roles in increased heat tolerance as well as freezing tolerance (Fu *et al.*, 1998). In potato, 15 d at 5/2 °C day/night increased both heat- and freezing-stress resistance in *Solanum commersonii* and other species capable of cold acclimation (Palta *et al.*, 1981). However, there is limited understanding of the molecular and biochemical mechanisms that confer enhanced heat tolerance in cold-acclimated plants.

Epigenetic regulation can play an important role in plant adaptation to abiotic stresses (Chinnusamy and Zhu, 2009). For instance, drought-induced expression of stress-responsive genes is associated with an increase in H3K4 trimethylation and H3K9 acetylation in *Arabidopsis* (Kim *et al.*, 2008). In tobacco, aluminum, paraquat, salt, and cold stresses have been found to induce DNA demethylation in the coding sequence of the *NtGPDL* gene (Choi and Sano, 2007). In addition, previous experiments have shown that cold acclimation can change DNA methylation levels in *Cannabis sativa* (Mayer *et al.*, 2015). Alterations in DNA methylation in *Celtis bungeana* have also been found to occur over periods of chilling and freezing (Song *et al.*, 2015). All of these results indicate that epigenetic changes, such as DNA methylation or demethylation, may occur in cold-acclimated plants.

In plant genomes, DNA methylation can occur either symmetrically at cytosines in both CG and CHG (H = A, T, or C) contexts or asymmetrically in a CHH context (Vanyushin and Ashapkin, 2011). Methylated DNA immunoprecipitation sequencing (MeDIP-seq) is a cost-effective method for studying whole-genome DNA methylation based on immunoprecipitation. The MeDIP-seq approach employs an antibody against 5-methylcytosine or methyl-binding domain proteins to capture methylated DNA, which is subsequently subjected to next-generation sequencing (Zhao *et al.*, 2014). Bok choy (also known as pak choi), which is a variety of Chinese cabbage (*Brassica rapa* ssp. *chinensis* L.) without heads, is an important vegetable in the middle and lower Yangtze region of China and in other Asian countries (http://nhccbase.njau.edu.cn/website/). In our previous research, we found that cold-acclimated bok choy also displayed increased heat tolerance and a high growth rate. To better understand the molecular and biochemical mechanisms of cross-adaptation that confer enhanced heat tolerance to cold-acclimated bok choy, in this study we characterized genome-wide DNA methylation patterns in control and cold-acclimated bok choy leaves using MeDIP-seq. Genetic validation and treatment with the DNA methylation inhibitor 5-aza-2-deoxycytidine (Aza) were used to study the causal link between changes in DNA methylation and gene expression on the one hand and physiological changes on the other hand. Physiological and molecular analyses were used to obtain a thorough understanding of the regulation of cross-adaptation. In addition, the function of the candidate gene *BramMDH1* is further discussed.

## Materials and methods

### Plant material and growth conditions

‘NHCC004’ is a cold-acclimated bok choy (*B. rapa* ssp. *chinensis* L.) cultivar and was used for the experiments. Plants were grown in pots containing a soil:vermiculite mixture (3:1) in the greenhouse of Nanjing Agricultural University in China, and the controlled-environment growth chamber maintained cycles of 16 h of light (approximately 300 μmol photons m^–2^ s^–1^) at 23 °C and 8 h of dark at 18 °C. For cold acclimation (CA) treatment, 40-d-old plants were transferred for an additional 2 weeks to a 4 °C growth cabinet under a 16-h day-length at 150 μmol photons m^–2^ s^–1^, then transferred back to greenhouse conditions for 1 week of recovery. For the control (CK) treatment, 40-d-old plants were transferred to a low-light (150 μmol photons m^–2^ s^–1^) chamber for 3 d at 16 h/23 °C light, 8 h/18 °C dark, then transferred back to greenhouse conditions for 1 week of recovery. After treatment, the third fully expanded leaf from the top of the plant was collected, frozen in liquid nitrogen, and stored at −80 °C for subsequent analysis.

For 5-aza-2-deoxycytidine (Aza) treatment, *B. rapa* seeds were germinated on Linsmaier and Skoog (LS) medium (Caisson Labs) plates containing 20 μM Aza (Sigma-Aldrich) and were grown for 15 d before measurement.

### DNA preparation and MeDIP-seq

DNA from leaves of CK and CA plants was isolated using a Universal Genomic DNA Extraction Kit (TaKaRa, Japan). About 50 ng per sample of purified DNA was then sent to the Beijing Genomics Institute (BGI, Shenzhen, Guangdong, China) for MeDIP-seq analysis by Illumina HiSeq 2000 (Illumina Inc., CA, USA). Data filtering included removing adapter sequences, contamination, and low-quality reads from among the raw reads. All 49-bp clean reads were mapped to the *B. rapa* genome [downloaded from the *Brassica* database (BRAD) version 1.5; http://brassicadb.org/brad/]. Only unique alignments with no more than two mismatches were considered for further analysis by SOAP2.21 (http://soap.genomics.org.cn). Whole-genome peak scanning was based on a defined analysis model using MACS 1.4.0 (http://liulab.dfci.harvard.edu/MACS/) with a cut-off *P*-value of 1 × 10^–4^ to exclude false-positive peaks or noise. Peaks of CK and CA samples were merged as candidate differentially methylated regions (DMRs) using MACS 1.4.0 (*P*-value ≤0.01 and at least a 2-fold change in sequence counts). For each candidate DMR, the number of reads for each sample was calculated. Then, numbers of reads were assessed with chi-square statistics and false-discovery rate (FDR) statistics to identify true DMRs. Methylated regions were deemed significantly differentially methylated across CK and CA samples with a *P*-value <0.05, FDR <0.05, and at least a 2-fold change in sequence counts. Genes that were significantly differentially methylated (DMGs) were used for gene ontology (GO) analysis and KEGG (Kyoto Encyclopedia of Genes and Genomes) pathway analysis.

### Bisulfite sequencing PCR (BSP) analysis

Genomic bisulfite sequencing was performed to confirm DNA methylation levels. Genomic DNA was extracted from CK and CA cells using a Universal Genomic DNA Extraction Kit (Takara, Japan) according to the manufacturer’s instructions, and 500 ng genomic DNA was treated with sodium bisulfite using the EZ DNA Methylation-Gold Kit (Zymo Research, Orange, CA, USA). Primers, which were designed using the MethPrimer program (http://www.urogene.org/cgi-bin/methprimer/methprimer.cgi), are shown in Supplementary Table S1 at *JXB* online. Then, the BSP products were cloned into a pMD19-T simple vector (Takara, Japan) according to the manufacturer’s instructions. For each line and each gene, ten positive clones were randomly selected for subsequent sequencing. After this, the amplicon sequence data were aligned to the *B. rapa* reference genome, and the extent of methylation (methylation level) was calculated by dividing all CpGs analyzed by the total number of methylated CpGs detected.

### Analysis of gene expression

Leaf samples (500 mg) were ground in liquid nitrogen. RNA extraction and first-strand cDNA synthesis were performed according to the manufacturer’s instructions using the Qiagen RNeasy Kit and SuperScript III reverse transcriptase (Invitrogen). Data were collected at 72 °C in each cycle, and the expression levels of genes were calculated with iQ5 optical system software version 2.0 using *BraGAPDH* (Bra016729) as the reference gene. Quantitative RT-PCR analysis included three biological replicates. RT-PCR primer sequences are shown in Supplementary Table S1. Products of qPCR were sequenced for accuracy.

### Gas exchange parameters

The net photosynthetic rate (*P*_N_) and dark respiration rate (*R*_d_) of the fully expanded leaves (the third leaves from the top of the plant) were measured using a portable photosynthesis system (LI-6400, LI-COR Inc., USA). Leaf temperatures were maintained at 23 °C. Relative humidity in the assimilation chamber was maintained at 60–70%, the external CO_2_ concentration was maintained at 400 ± 10 μmol mol^–1^, and the light intensity was maintained at 1000 μmol photons m^–2^ s^–1^.

### Chlorophyll content, biomass, and organic acids measurements

Leaf chlorophyll (Chl) content was measured using a hand-held Chl meter (SPAD-502Plus, Minolta Corp., Spectrum Technologies, Inc.). The measurement points were randomly selected on the third fully developed leaves from the top of the plant, and three points were selected on each leaf (Richardson *et al.*, 2002).

For biomass analysis, leaves from the plant were harvested. The dry weight (DW) was determined after drying the leaves at 80 °C for 2 d.

For organic acids analysis, a 0.5-g leaf sample was collected and frozen in liquid nitrogen for organic acid extraction as described previously (Johnson *et al.*, 1996). The organic acids content was measured according to the method of Tesfaye *et al.* (2001) in 25 μl of extraction sample. All analyses included three independent replicates.

### Generation of transgenic Arabidopsis

To generate the minigene constructs (*pBramMDH1:HA-BramMDH1*, *pBraKAT2:HA-BraKAT2*, *pBraSHM4:HA-BraSHM4*, and *pBra4CL2:HA-Bra4CL2*) we first cloned the full-length cDNA into the pENTR/D-TOPO vector (Invitrogen) to make the gene fusion in-frame with the HA sequence (primer sequences are shown in Supplementary Table S1). Then, the *BramMDH1* (A06: 662305–664304), *BraKAT2* (A05: 5994734–5996733), *BraSHM4* (A04: 5303170–5305169), and *Bra4CL2* (A05: 17253035–17255034) promoter regions (primer sequences are shown in Supplementary Table S1) were cloned into the pENTR 5′-TOPO vector (Invitrogen). After the sequences were verified, both the HA cDNA and the gene promoter were transferred into the R4pGWB501 vector using multiple Gateway reactions to generate the *pBramMDH1:HA-BramMDH1*, *pBraKAT2:HA-BraKAT2*, *pBraSHM4:HA-BraSHM4*, and *pBra4CL2:HA-Bra4CL2* binary vectors. These constructs were introduced into Arabidopsis (Col-0 and *met1*) plants using *Agrobacterium tumefaciens*-mediated transformation (strain ABI) with the floral dip method (Clough and Bent, 1998). Primary transformants (seedlings) were screened on Linsmaier and Skoog (LS) medium (Caisson) supplemented with 10 μg mL^–1^ hygromycin (Sigma-Aldrich). Stable transgenic plants (T_3_) were used in the study.

To generate the *35S:HA-BramMDH1*/Col-0 lines, the full-length sequence of *BramMDH1* was inserted into the pEarleyGate201 vector with HA tag by Gateway LR recombination (Invitrogen). This construct was used to transform *A. tumefaciens* strain ABI and then Arabidopsis plants (Col-0) using *Agrobacterium*-mediated plant transformation via floral dip. Transgenic lines were selected on LS plates containing 16 μg mL^–1^ Basta. A T_3_ line was used for further analysis.

### Accession numbers

*Brassica rapa* accession numbers used in this study are from the *Brassica* database (http://brassicadb.org/brad/).

## Results

### Global mapping of DNA methylation

In total, 51 020 408 clean reads were acquired from the MeDIP-seq analysis of the CK and CA samples. Over 76% of the CK reads were mapped, and 30% of the CK reads were uniquely mapped to the *B. rapa* genome, while for the CA samples, 79% and 32% of reads were mapped and uniquely mapped, respectively (Table 1). Uniquely mapped reads were detected on all chromosomes (see Supplementary Fig. S1). When identifying global DNA methylation patterns, the number of methylated peaks detected by MeDIP-seq is important (Hu *et al.*, 2013). We obtained 19 001 and 19 589 methylated peaks in the CK and CA samples, respectively, covering approximately 10.19% and 10.47%, respectively, of the *B. rapa* genome (Supplementary Table S2).

**Table 1.** *Summary of MeDIP-seq Illumina data mapped to the* Brassica rapa *genome*

Sample	Total reads	Mapped reads	Mapping rate (%)	Unique mapped read	Unique mapping rate (%)
CK	51 020 408	39 271 101	76.97	15 721 494	30.81
CA	51 020 408	40 477 918	79.34	16 694 860	32.72

**Fig. 1.**
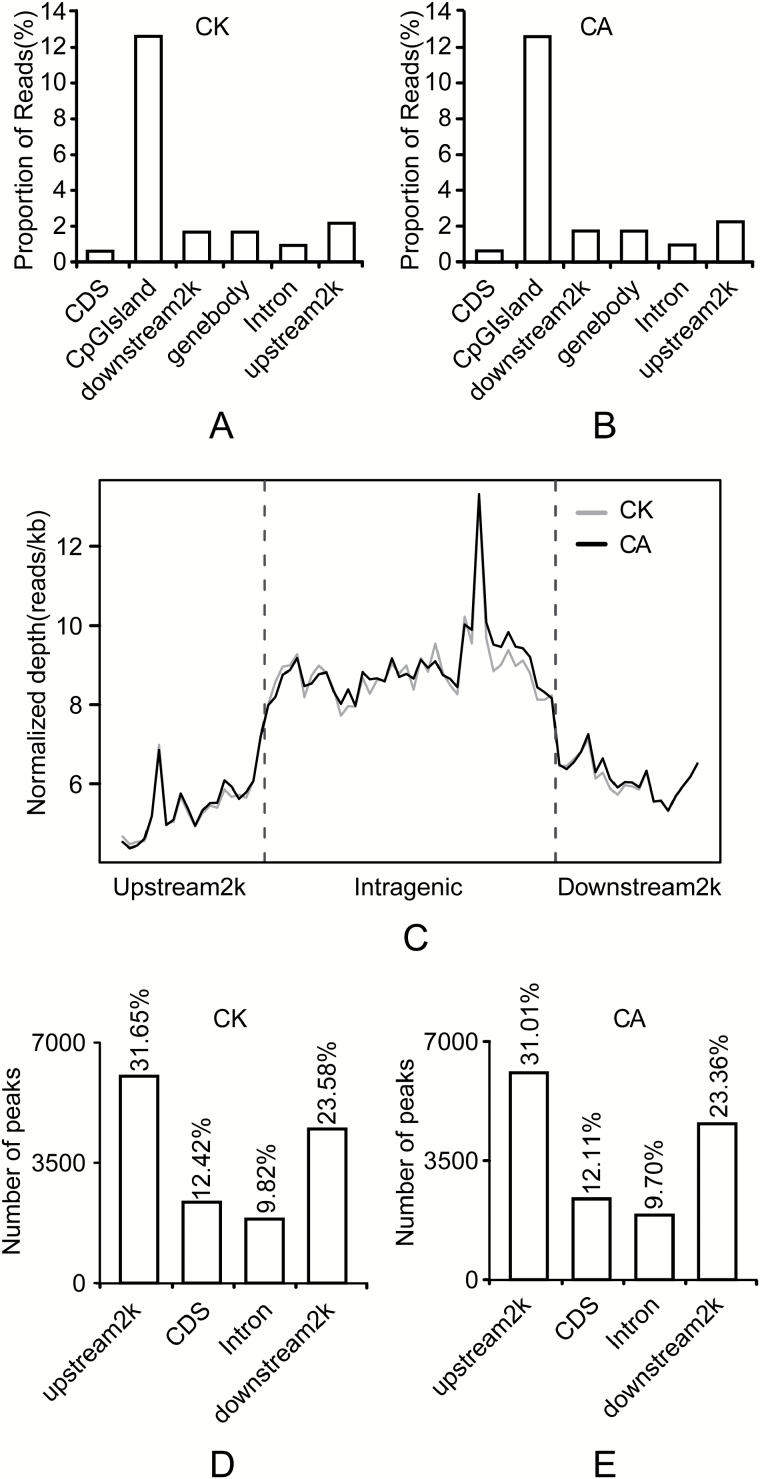
Distribution of uniquely mapped reads across different genomic regions. (A, B) Distribution of reads among CpG islands in CK (A) and CA (B). The *x*-axis shows the different gene regions and the *y*-axis shows the percentage of uniquely mapped reads. (C) Distribution of reads for the 2-kb region upstream of the transcription start site (TSS), the gene from the TSS to the transcription termination site (TTS), and the 2-kb region downstream of the TTS. DNA methylation was highest around the gene itself. (D, E) Distribution of DNA methylation peaks in different genomic regions in CK (D) and CA (E). The majority of peaks were present in the upstream2k region, followed by the downstream2k region, whereas the CDS and intron had fewer peaks. The *x*-axis shows the different genomic regions and the *y*-axis shows the number of peaks.

Different genomic regions exhibited different methylation patterns. The majority of reads were mapped to CpG islands, followed by reads that mapped to the 2 kb region upstream of genes (upstream2k) (Fig. 1A, B). A depletion of, or increase in, reads was often observed within the gene coding sequences in the CA samples. In contrast, a gradual decrease in reads occurred across the upstream2k region (Fig. 1C). The distribution of reads on the genome (recorded in a 1000-bp window) had a peak at 20–25 CpG in both samples (see Supplementary Fig. S2A, B). However, the distribution of peaks in the window was maximum at 15–20 CpG in both samples (Supplementary Fig. S2C, D). The distributions revealed that most of the reads and peaks tended to be in the regions with low numbers of CpG. More interestingly, reads distributed in satellites only appeared in the CA samples, although only low numbers were detected (Supplementary Fig. S2E, F).

Methylation peaks, referred to as methyl-cytosine-enriched regions, are important for the identification of global DNA methylation patterns (Hu *et al.*, 2013). In our study, peaks were most prevalent in the upstream2k regions in both the CK and CA samples (31.65% and 31.01%, respectively), followed by those within the region 2 kb downstream (downstream2k) of the transcription termination site (TTS) and coding DNA sequence (CDS) of the transcription start site (TSS); introns exhibited relatively fewer peaks (Fig. 1D, E). Methylation of CpG islands in the promoter and CDS regions is known to be involved in the regulation of gene expression, and these regions are reported to be hypomethylated in the vertebrate genome (Jones, 2012).

**Fig. 2.**
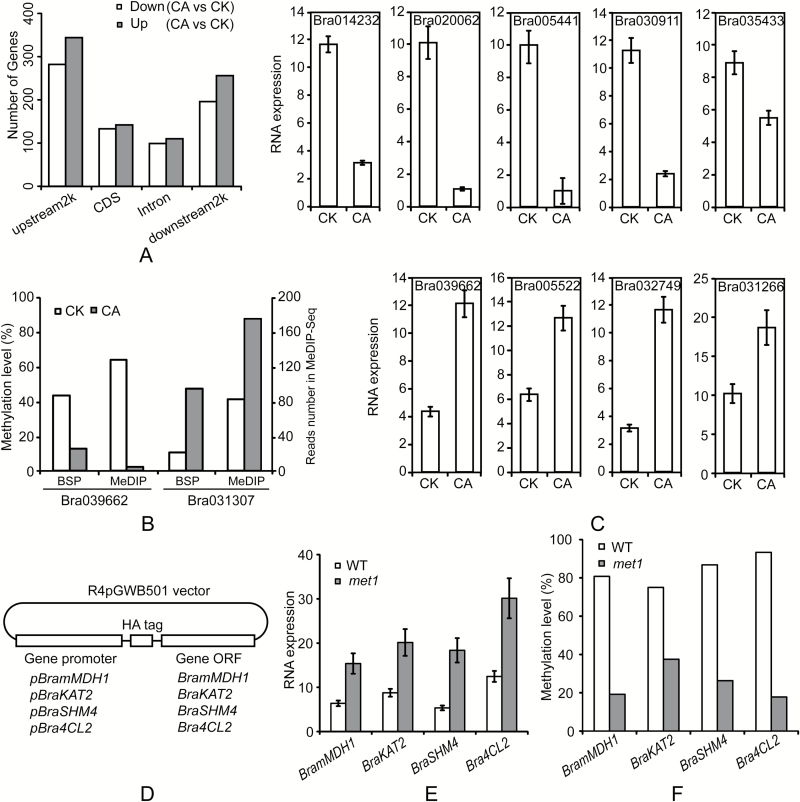
Differentially methylated genes between CK and CA plants. (A) Distribution of differentially methylated genes (DMGs) across different genomic regions. The number of differentially methylated genes is given in Table 2. (B) Validation of MeDIP-seq data by bisulfite sequencing (BSP). Two highly methylated regions obtained from MeDIP-seq data were randomly selected for verification by BSP. The left *y*-axis left shows the extent of methylation calculated by dividing all of the CpGs analyzed by the total number of methylated CpGs detected, and the right *y*-axis shows the methylation level (read number) according to MeDIP-seq data. (C) mRNA expression of genes that exhibited down-methylation in the upstream2k between CK and CA plants. All significantly differentially methylated genes in upstream2k between CK and CA are down-methylated (see GO and KEGG results, *P*<0.05, Supplementary Tables S4–S7). From the 39 genes with differential methylation in upstream2k between CK and CA, we found only nine genes showing significantly different expression (five low-expression in CA are shown in the top row and four high-expression in CA are shown in bottom row). Vertical bars represent the standard deviation (SD) of the mean of three biological replicates. (D) Schematic drawing of the minigenes used in Arabidopsis transformation. (E) qRT-PCR analysis of transcript abundances of *BramMDH1*, *BraKAT2*, *BraSHM4*, and *Bra4CL2* minigenes in wild-type and *met1* Arabidopsis. Vertical bars represent the SD of the mean of three biological replicates. (F) DNA methylation levels of *BramMDH1*, *BraKAT2*, *BraSHM4*, and *Bra4CL2* minigenes in wild-type and *met1* Arabidopsis.

A comparison of DMRs revealed a total 29 624 between the CK and CA samples (see Supplementary Table S3). Next, we identified genes containing DMRs in both groups, resulting in a total of 1562 DMGs in the CA samples. These included 626 that were differentially methylated in the upstream2k (282 genes with decreased methylation, 344 genes with increased methylation), 275 in the CDS (133 genes with decreased methylation, 142 genes with increased methylation), 209 in the intron (99 genes with decreased methylation, 110 genes with increased methylation), and 452 in the downstream2k (196 genes with decreased methylation, 256 genes with increased methylation). More genes were up-methylated (*n* = 852) than down-methylated (*n* = 710) in CA plants compared to those in CK plants (Fig. 2A, Table 2).

**Table 2.** Numbers of differentially methylated genes across different gene regions

CA/CK	upstream2k	CDS	Intron	downstream2k
Down	282	133	99	196
Up	344	142	110	256

### Biological features of genes that exhibit differential methylation

The 1562 DMGs identified between the CK and CA samples were assigned to terms in the Gene Ontology (GO) and Kyoto Encyclopedia of Genes and Genomes (KEGG) databases. Using the DAVID program (https://david.ncifcrf.gov/), we performed GO analysis. GO assignments revealed that genes with increased methylation in the CA samples were significantly involved in abscisic acid glucosyltransferase activity (terms for downstream2k, *P*<0.05, Supplementary Table S4). Genes with decreased methylation were strongly enriched in two categories: GTPase activity and L-malate dehydrogenase activity (terms for upstream2k, *P*<0.05, Supplementary Table S5). To determine the significant pathways involved in differential methylation, we used the KEGG pathway database to predict putative functions. Genes with increased methylation in the CA samples were significantly enriched in two pathways: protein export (terms for downstream2k, *P*<0.05, Supplementary Table S6) and homologous recombination (terms for introns, *P*<0.05, Supplementary Table S6). Genes with decreased methylation were related to five pathways, namely phagosome, citrate cycle (TCA cycle), carbon fixation in photosynthetic organisms, biosynthesis of secondary metabolites, and pyruvate metabolism (terms for upstream2k, *P*<0.05, Supplementary Table S7). Genes with methylation peaks in both the promoter and CDS regions were considered to be methylated genes (Song *et al.*, 2015). The functional classification of DMGs revealed that malate dehydrogenase activity and carbon fixation were down-methylated in the upstream2k regions of the CA samples compare to those of the CK samples (Supplementary Tables S4–S7).

To validate the MeDIP-seq data, two regions (Bra031307, up-methylation in downstream2k, Chr A5: 16 837 978–16 839 977; Bra039662, down-methylation in upstream2k, Chr A6: 662 305–664 304) were selected for bisulfite sequencing. The results obtained for the two gene regions were in accordance with the MeDIP-seq results (Fig. 2B).

To determine whether DNA methylation affects gene expression, we selected all 39 genes with differential methylation in the upstream2k and performed qPCR in CK and CA plants (gene lists are shown in the GO and KEGG results, *P*<0.05, Supplementary Tables S4–S7). A total of nine DMGs exhibited differential expression patterns between CK and CA plants: four were up-regulated and five were down-regulated in CA plants (Fig. 2C). The remaining genes exhibited no significant difference in expression between the samples (data not shown). The qPCR results indicated that promoter methylation does not necessarily affect expression. These data highlight the importance of the down-methylation of *BramMDH1* (Bra039662), *BraKAT2* (Bra005522), *BraSHM4* (Bra032749), and *Bra4CL2* (Bra031266) during cold acclimation.

### Genetic validation reveals that DNA methylation affects gene expression

Methylation levels in promoter regions generally correlate with gene expression levels (Zilberman *et al.*, 2007). The maintenance of DNA methylation requires the DNA methyltransferase MET1 (methyl transferase 1), as well as the SWI/SNF2-like chromatin-remodeling protein DDM1 (decrease in DNA methylation 1) (Jeddeloh *et al.*, 1999). Since mutants in the *B. rapa* background were unavailable, to confirm that the changes in DNA methylation had caused the changes in gene expression in *BramMDH1*, *BraKAT2*, *BraSHM4*, and *Bra4CL2*, we transformed the minigenes (Fig. 2D) *pBramMDH1:HA-BramMDH1*, *pBraKAT2:HA-BraKAT2*, *pBraSHM4:HA-BraSHM4*, and *pBra4CL2:HA-Bra4CL2* into wild-type (WT) Arabidopsis and into a *met1* mutant defective in DNA methylation. We first determined whether the expression levels of the minigenes were altered in the *met1* mutant. Indeed, minigene expression levels were significantly higher in the *met1* mutant than in the WT (Fig. 2E). Then, to demonstrate an association between DNA methylation and transcriptional expression, we determined the promoter methylation levels of the minigenes in the WT and mutant lines. BSP results showed that the methylation levels of minigenes were generally higher in the WT than in the *met1* mutant (Fig. 2F). Together, these results suggest that DNA methylation of promoter regions is responsible for the altered expression of *BramMDH1*, *BraKAT2*, *BraSHM4*, and *Bra4CL2*.

### Cold-acclimated *B. rapa* shows enhanced heat tolerance and increased biomass

**Fig. 3.**
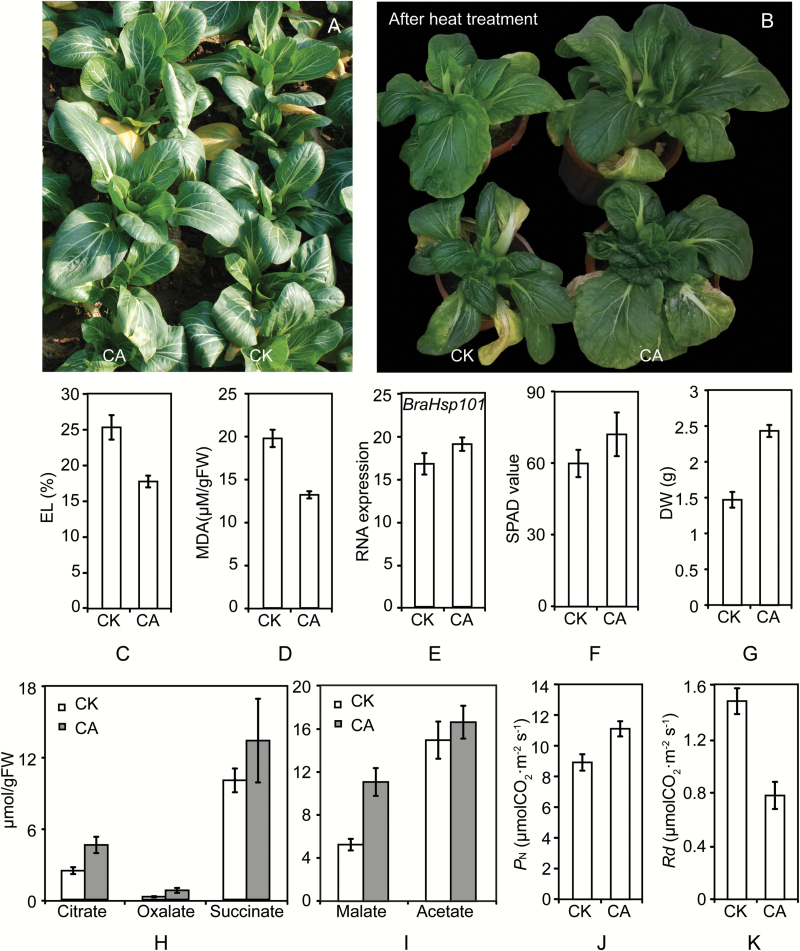
Cold-acclimated *Brassica rapa* shows elevated heat tolerance and high growth rate. Relative growth (A), heat tolerance (B), levels of electrolyte leakage (EL) (C), and malonaldehyde (MDA) contents (D) of CA and CK plants. (E) mRNA expression of the heat-shock protein gene *BraHsp101* (Bra015922) in CK and CA plants. (F) Chlorophyll contents in CA and CK leaves. (G) Dry weight of CA and CK leaves. (H, I) Organic acid contents in leaves from CK and CA plants. (J) Net photosynthesis (*P*_N_) and (K) dark respiration (*R*_d_) values in CA and CK plants. Error bars show the standard deviation (*n* = 3). (This figure is available in colour at *JXB* online).

Heat stress is a major abiotic factor limiting the growth of temperate plant species in many areas during summer months, and it may present a major challenge as global warming continues. Interestingly, when we planted CK and CA plants in the field, we found that the CA plants exhibited higher growth rates compared to those of CK plants in the summer (Fig. 3A). To confirm that the CA plants had elevated heat tolerances, CK and CA plants were grown continuously in controlled environment chambers at 40/35 °C for 12 h and then transferred to 23/18 °C conditions for 1 week of recovery. CA plants indeed displayed enhanced heat tolerance (Fig. 3B). Next, electrolyte leakage (EL) and malonaldehyde (MDA) content were measured in leaves of CK and CA plants. CA leaves exhibited significantly lower EL and MDA values compared with those of CK leaves (Fig. 3C, D), indicating that CA plants have enhanced heat tolerance compared to that of CK plants.

Heat-stress factors (Hsf) and heat-shock proteins (Hsp) are central control proteins in the heat-stress response (Schöffl *et al.*, 1998). *HsfA2* is a major Hsf (Nishizawa *et al.*, 2006; Schramm *et al.*, 2006) in the plant heat stress response. In the HSF-HSP-HSBP1 pathway, *HsfA1a* triggers the heat-stress response by inducing *HsfA1b* and *HsfA2* expression, which induces the expression of various Hsp proteins. *Hsp70*, *Hsp101*, and *sHsp* participate in the repair of damaged proteins (Qu *et al.*, 2013). To further study the mechanisms of enhanced heat tolerance in the CA plants, we analyzed the transcript levels of *BraHsfA2* (Bra000557), *BraHsp70* (Bra006027), and *BraHsp101* (Bra015922) in CK and CA plants by qPCR. CA plants exhibited a small increase in *BraHsp101* mRNA, but this was not statistically significant (Fig. 3E). Transcript levels of *BraHsfA2* and *BraHsp70* did not appear to be affected by cold acclimation (data not shown). These results demonstrate that enhanced heat tolerance in CA plants is not caused by higher expression of Hsf and Hsp proteins.

In CA plants, a high growth rate was observed (Fig. 3A), so we measured chlorophyll content and biomass in CA leaves. CA leaves exhibited a small increase in chlorophyll content, but this was not significant (Fig. 3F). However, the DW of CA leaves was markedly elevated (Fig. 3G). Together with the functional classification of DMGs, which revealed that carbon fixation genes were differentially methylated in CA plants (see Supplementary Tables S4–S7), these results suggest that CA plants may exhibit enhanced photosynthesis or assimilation abilities, which serve to increase plant biomass.

### Cold-acclimated plants exhibit increases in organic acids and photosynthesis

Heat stress induces changes in various metabolites, such as organic acids, amino acids, and carbohydrates, which have important functions in photosynthesis and respiration (Merewitz *et al.*, 2012). Primary metabolic profiling has revealed that organic acids are affected by heat treatment in citrus (Yun *et al.*, 2013). Citric acid, as a vital organic acid, has been reported to be closely related to aluminum poisoning (Tesfaye *et al.*, 2001; Ma and Furukawa, 2003), iron stress (Shlizerman *et al.*, 2007), heavy metal stress tolerance (Gao *et al.*, 2010), and salinity stress (Sun and Hong, 2011). Exogenous citric acid improves heat stress tolerance in tall fescue (Hu *et al.*, 2016). To investigate if the enhanced heat tolerance of CA plants was linked to an increase in organic acids, we measured the organic acid contents of the leaves of CK and CA plants. Leaves from CA plants showed increased organic acid concentrations compared with those of leaves from CK plants (Fig. 3H, I). Citrate, oxalate, and malate accumulation in CA leaves were about 2-fold higher than in CK plants, while succinate and acetate were not significantly different between CA and CK leaves. These findings suggest that the elevated organic acids in CA plants may contribute to enhanced heat resistance.

As already mentioned, CA plants had higher growth rates and biomass (Fig. 3A, G). To confirm that the higher growth rate in CA plants and higher levels of photosynthesis or assimilation were linked, we measured the *P*_N_ and *R*_d_ values of the fully expanded leaves in CA and CK plants. *P*_N_ was 25% higher in CA leaves than in CK leaves (Fig. 3J), while *R*_d_ in CA leaves was nearly half of that in CK leaves (Fig. 3K). Therefore, the high *P*_N_ and low *R*_d_ of CA leaves enhances net photosynthesis, which plays a critical role in the net growth rate of the plants.

### DNA methylation inhibitor promotes growth but not heat tolerance in *B. rapa*

**Fig. 4.**
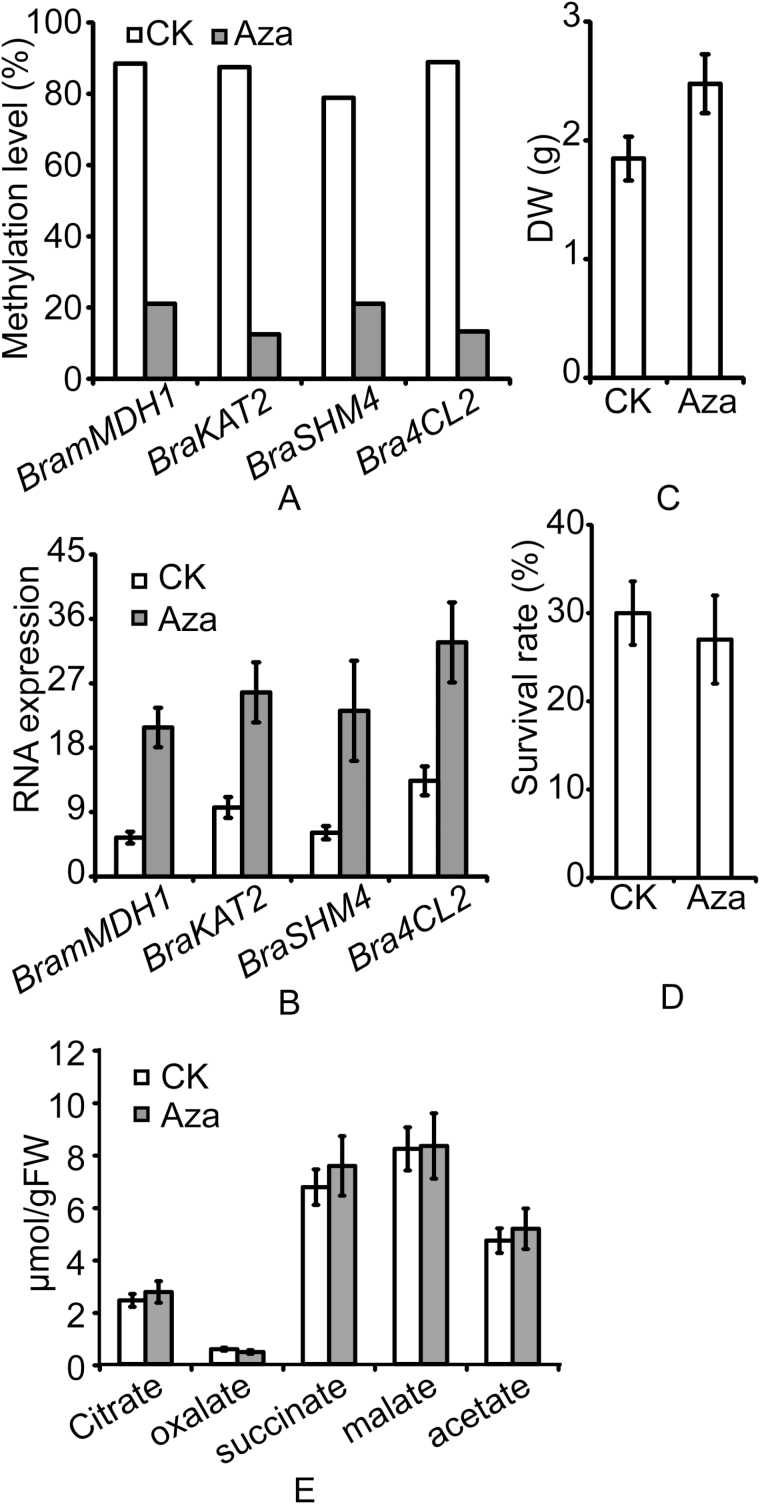
Effects of DNA methyltransferase inhibitor (5-aza-2-deoxycytidine, Aza) on *Brassica rapa* seedlings. (A) DNA methylation levels of *BramMDH1*, *BraKAT2*, *BraSHM4*, and *Bra4CL2* in untreated (CK) and Aza-treated seedlings. (B) qRT-PCR analysis of transcript abundances of *BramMDH1*, *BraKAT2*, *BraSHM4*, and *Bra4CL2* in untreated and Aza-treated seedlings. (C) Dry weight of leaves from untreated and Aza-treated seedlings. (D) Survival rates of untreated and Aza-treated seedlings after heat shock. (E) Organic acid contents in untreated and Aza-treated seedlings. Error bars show the standard deviation of the mean of three biological replicates.

MeDIP-seq data showed that cold acclimation induced both up- and down-regulation of methylation. However, all four candidate genes experienced promoter demethylation during cold acclimation. We sought to determine whether this DNA demethylation was the main reason for the increased heat tolerance and higher growth rate of CA plants. To further investigate whether DNA demethylation affected the expression of *BramMDH1*, *BraKAT2*, *BraSHM4*, and *Bra4CL2* and the physiological phenotype of *B. rapa*, plants were treated with Aza, a commonly used DNA methylation inhibitor (Goffin and Eisenhauer, 2002; Zhong *et al.*, 2013). The methylation and expression levels of the four candidate genes were measured by BSP and qPCR. As expected, Aza treatment led to decreased DNA methylation levels in the candidate genes (Fig. 4A). Compared to untreated plants, Aza treatment led to enhanced transcription of *BramMDH1*, *BraKAT2*, *BraSHM4*, and *Bra4CL2* (Fig. 4B). Together with MeDIP-seq data and genetic validation, therefore, we can conclude that the altered expression patterns of the genes can be attributed to the lower levels of DNA methylation.

In addition, the heat tolerances and growth rates of untreated and Aza-treated *B. rapa* were assessed by measuring seedling survival rates and DW, respectively, after heat stress. Aza treatment led to increased biomass in *B. rapa* (Fig. 4C). However, no significant difference in survival rate was observed after heat shock of untreated and Aza-treated *B. rapa* (Fig. 4D). Since we had observed that an increase in organic acids in CA plants may contribute to their enhanced heat resistance, we hypothesized that there would be no increase in organic acids in Aza-treated *B. rapa*. As expected, organic acids were not elevated in Aza-treated *B. rapa* compared with levels in untreated plants (Fig. 4E). As Aza is a DNA methylation inhibitor, these results suggest that DNA demethylation alone is not sufficient to increase plant heat tolerance.

### Over-expression of *BramMDH1* in Arabidopsis leads to increased heat tolerance and growth rate

**Fig. 5.**
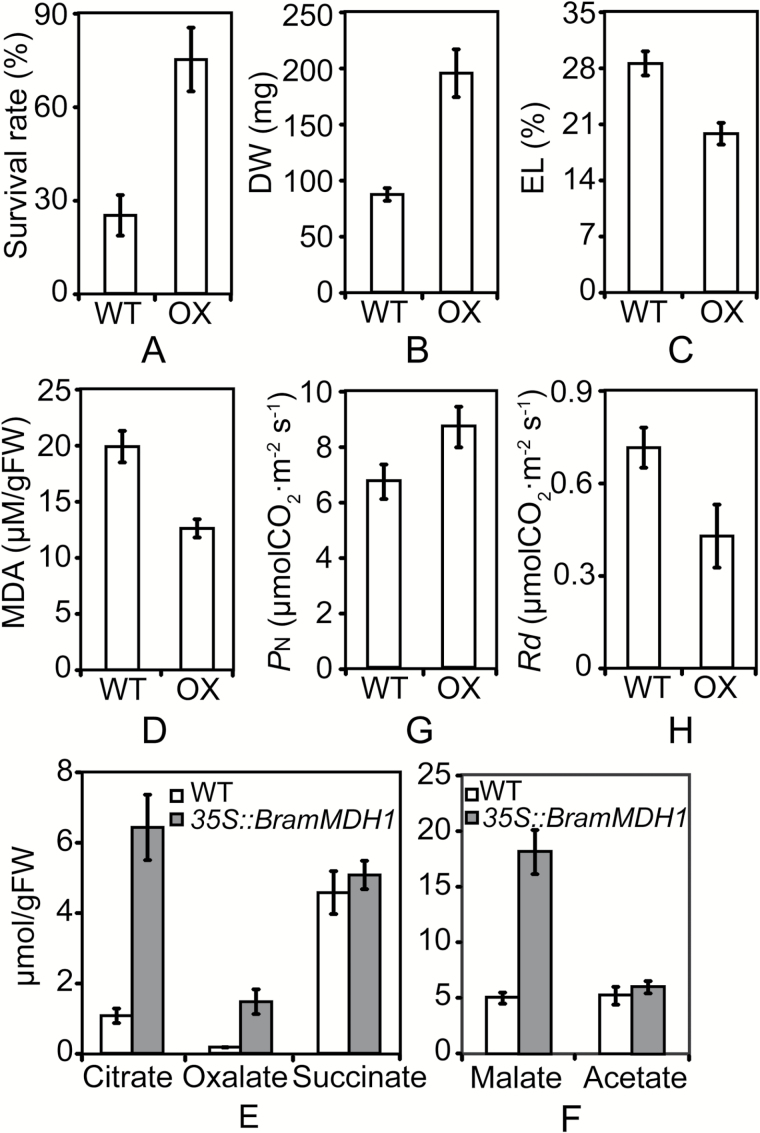
Over-expression of *BramMDH1* in Arabidopsis results in increased heat tolerance and higher growth rate. (A) Survival rate of *35S::BramMDH1* (OX) and wild-type (WT) Arabidopsis after heat shock. (B) Dry weight, (C) electrolyte leakage (EL) levels, and (D) malonaldehyde (MDA) contents of *35S::BramMDH1* (OX) and WT Arabidopsis. (E, F) Organic acid contents in leaves of *35S::BramMDH1* and WT Arabidopsis. (G) Net photosynthesis (*P*_N_) and (H) dark respiration (*R*_d_) in *35S::BramMDH1* and WT Arabidopsis. Error bars show the standard deviation (*n* = 3).

Among the four candidate genes, only *BramMDH1* is reported to be associated with organic acids, leaf respiration, plant growth, and aluminum tolerance (Journet *et al.*, 1981; Tesfaye *et al.*, 2001; Tomaz *et al.*, 2010). To confirm that *BramMDH1* was causally linked to increased heat tolerance and growth rate, we produced transgenic Arabidopsis expressing *BramMDH1* cDNA under the control of the constitutive cauliflower mosaic virus 35S promoter. Levels of *BramMDH1* mRNA were analyzed in selected transgenic and WT plants by qPCR. The *35S::BramMDH1* transgenic Arabidopsis exhibited up to a 5-fold increase in *BramMDH1* transcription compared with that of the WT (see Supplementary Fig. S3). Next, we investigated whether overexpression of *BramMDH1* led to increased heat tolerance and growth rate. As expected, *35S::BramMDH1* exhibited enhanced heat tolerance and biomass (Fig. 5A, B). Survival rate after heat shock at 40 °C for 1 h was 3-fold higher in *35S::BramMDH1* than in the WT (Fig. 5A). In addition, *35S::BramMDH1* had lower EL and MDA values than those of the WT after heat stress (Fig. 5C, D). Moreover, the DW of *35S::BramMDH1* was also elevated (Fig. 5B). Consistent with enhanced heat tolerance and higher growth rate, significant increases in organic acids (Fig. 5E, F) and *P*_N_ (Fig. 5G) and a decrease in *R*_d_ (Fig. 5H) were observed in *35S::BramMDH1*. Together with the above results, these findings suggest that *BramMDH1* plays an important role in enhanced heat tolerance and growth rate.

Like *35S::BramMDH1*, cold-acclimated *B. rapa* exhibited elevated *BramMDH1* transcription, which was able to increase plant heat tolerance. However, Aza-treated *B. rapa*, which also exhibited high *BramMDH1* expression (Fig. 4B), showed no significant increase in heat tolerance (Fig. 4D) and no elevation in organic acids (Fig. 4E). Both cold-acclimated and Aza-treated *Brassica rapa* had a high level of transcription of *BramMDH1* that positively contributed to plants heat tolerance. But only cold-acclimated *Brassica rapa* had elevated heat tolerance. Therefore, as both methylation and demethylation were found in CA plants, we speculate that DNA methylation acts to suppress the expression of negative genes for organic acids and that it is also needed to increase heat tolerance in CA plants.

## Discussion

Previously, many reports have demonstrated that cold acclimation acts to increase cold tolerance in many organisms, such as Arabidopsis (Wanner and Junttila, 1999; Thomashow, 1999), tomato (Barrero-Gil *et al.*, 2016), rice (Ahamed *et al.*, 2012), wheat (Hurry *et al.*, 1995), *Chorispora bungeana* (Song *et al.*, 2015), *C. sativa* (Mayer *et al.*, 2015), and *Drosophila melanogaster* (MacMillan *et al.*, 2016). Interestingly, we observed that cold-acclimated bok choy cabbage also exhibited enhanced heat tolerance and higher growth rate (Fig. 3A, B). Exposure of plants to a moderate stress can induce resistance to other stresses in a process called cross-adaptation, which has been demonstrated for several stress combinations (Sabehat *et al.*, 1998; Bowler and Fluhr, 2000). For example, heat-shock treatment can not only improve plants’ thermotolerance but also increase their tolerance to other stresses, such as chilling (Collins *et al.*, 1995; Sabehat *et al.*, 1996; Kadyrzhanova *et al.*, 1998), salinity (Kuznetsov *et al.*, 1993), drought (Kuznetsov *et al.*, 1999), and metal ion stress (Orzech and Burke, 1988; Neumann *et al.*, 1994).

Epigenetic regulation plays an important role in plant adaptation to abiotic stresses (Chinnusamy and Zhu, 2009). Therefore, to investigate the molecular and biochemical mechanisms of cross-adaptation that confer elevated heat tolerance in cold-acclimated bok choy, we performed a comprehensive analysis of DNA methylation changes in leaves from control (CK) and cold-acclimated (CA) plants. Our analysis identified 29 624 regions and 1562 unique genes (852 genes with increased methylation, 710 genes with decreased methylation) that exhibit differential DNA methylation in CA plants compared to levels in CK plants (Fig. 2A; Table 2; Supplementary Table S3). These results indicate that both DNA methylation and demethylation occur during cold acclimation. Similar results were found in recent studies in *C. sativa* (Mayer *et al.*, 2015) and *C. bungeana* (Song *et al.*, 2015). Our methylome data show that cold acclimation induces both up- and down-regulation of methylation, suggesting a complex regulation of methylation involving various methylating and possibly demethylating agents. In summary, our experiments clearly demonstrate a significant role of changes in DNA methylation in cold acclimation.

Genes with differential methylation of both the promoter and gene regions were considered as DMGs (Hu *et al.*, 2013). We found that nearly 60% of DMGs exhibited differential methylation levels in the upstream2k and CDS regions in CA plants (Fig. 2A; Table 2). After GO and KEGG functional classification of DMGs from the upstream2k and CDS regions, we identified significant enrichment of DMGs in CA plants in pathways linked to GTPase and L-malate dehydrogenase activity (down-methylation in upstream2k, *P*<0.05, Supplementary Table S5), phagosome, citrate cycle (TCA cycle), carbon fixation in photosynthetic organisms, biosynthesis of secondary metabolites, and pyruvate metabolism (down-methylation in upstream2k, *P*<0.05, Supplementary Table S7). Overall, our DNA methylation profiles revealed that malate dehydrogenase activity and carbon fixation were significantly affected in CA plants. Hypermethylation of certain genomic regions may lead to suppressed transcription (Bird, 2007). In contrast, hypomethylation may lead to increased transcription. By combining genetic validation (Fig. 2E, F) and DNA methylation inhibitor data (Fig. 4A, B), we identified four candidate genes (*BramMDH1*, *BraKAT2*, *BraSHM4*, and *Bra4CL2*) that exhibited decreased DNA methylation in promoter regions and increased gene expression in CA plants.

Leaf EL and MDA levels are generally used to assess the extent of membrane damage caused by environmental stress (Cen *et al.*, 2016; Hu *et al.*, 2016). To further validate the enhanced heat tolerance in CA plants, we detected EL and MDA levels in leaves of CK and CA plants after heat stress. Consistent with the elevated heat tolerance in CA plants (Fig. 3B), CA leaves exhibited lower EL and MDA values compared with those in CK leaves (Fig. 3C, D). Next, to identify factors that may contribute to enhanced heat tolerance in CA plants, we tested if there were significant associations between elevated heat tolerance and the expression levels of Hsf and Hsp genes, which are involved in resistance to heat stress in plants (Schöffl *et al.*, 1998). Unexpectedly, we found no significant increases in the expression of Hsf and Hsp genes in CA plants compared with CK levels (Fig. 3E). This result indicated that enhanced heat tolerance in CA plants was not caused by elevated expression of Hsf and Hsp proteins, which is consistent with a previous report (Fu *et al.*, 1998). Many studies have suggested that organic acids are closely associated with abiotic stresses, such as aluminum poisoning (Ma and Furukawa, 2003), iron stress (Shlizerman *et al.*, 2007), heavy metal stress tolerance (Gao *et al.*, 2010), and salinity stress (Sun and Hong, 2011). We therefore tested for an association between elevated heat tolerance and organic acid content in CA plants. Indeed, significant accumulation of citrate, oxalate, and malate were observed in CA leaves (Fig. 3H, I), suggesting that an increase in organic acids in CA plants may contribute to enhanced heat resistance.

We observed a higher growth rate in CA plants. Additionally, DMGs were significantly enriched in malate dehydrogenase activity and carbon fixation in CA plants, pathways which have also been identified as associated with cold acclimation in Arabidopsis (Stitt and Hurry, 2002). Similarly, the primary components of photosynthesis, including thylakoid electron transport and the carbon reduction cycle, are affected by cold temperatures in many species, including tomato (Martin *et al.*, 1981), maize (Kingston-Smith *et al.*, 1997), and cucumber (Choi *et al.*, 1994). Moreover, following long-term cold-hardening of winter and spring cultivars of wheat and rape, winter cultivars had higher net assimilation rates and higher photosynthetic rates than the corresponding spring cultivars (Hurry *et al.*, 1995). Therefore, to identify factors that may contribute to high growth rates in cold-acclimated bok choy, we measured the chlorophyll content, *P*_N_, and *R*_d_, which play critical roles in photosynthesis and carbon fixation. We found no significant differences in chlorophyll content between CK and CA leaves (Fig. 3F). However, consistent with the fact that the CA plants have a higher growth rate, an increase in *P*_N_ and decrease in *R*_d_ were observed in CA leaves compared with values in CK leaves (Fig. 3J, K). These results indicate that enhanced photosynthesis in CA plants may contribute to their higher growth rate.

CA plants exhibited elevated organic acids and enhanced photosynthesis, which may contribute to increased heat tolerance and higher growth rate, respectively. We therefore sought to determine whether any of the four candidate genes were responsible for the elevated organic acids and enhanced photosynthesis. Recent studies have shown that *mMDH1* in Arabidopsis plays an important role in plant growth rate, respiration, and photosynthesis. The slow-growing *mmdh1mmdh2* double-mutant exhibits an elevated leaf respiration rate. Complementation of *mmdh1mmdh2* with *mMDH* cDNA suppressed the respiration rate and increased plant growth (Tomaz *et al.*, 2010). In addition, overexpression of malate dehydrogenase in transgenic alfalfa enhances organic acid synthesis and confers tolerance to aluminum (Tesfaye *et al.*, 2001). In CA plants, we found promoter demethylation led to elevated *BramMDH1* expression. However, it was still unclear whether the elevated expression of *BramMDH1* in CA plants had a direct effect on enhanced heat tolerance and/or growth rate. To determine the role of *BramMDH1* in heat tolerance, we overexpressed *BramMDH1* in Arabidopsis. After heat stress, *35S::BramMDH1* exhibited a higher survival rate and lower EL and MDA values when compared with those of the WT (Fig. 5A, C, D). In addition, elevated levels of organic acids were found in leaves from *35S::BramMDH1* plants compared with levels in controls (Fig. 5E, F). Moreover, *35S::BramMDH1* displayed increased *P*_N_ (Fig. 5G) and decreased *R*_d_ (Fig. 5H) values, consistent with the higher DW (Fig. 5B) of *35S::BramMDH1* plants. These experimental results are in agreement with our bok choy data, where CA plants showed enhanced heat tolerance and higher growth rate in parallel with increased expression and reduced DNA methylation of *BramMDH1*. Hence, our functional data support an active role of the candidate gene *BramMDH1* in enhanced heat tolerance and higher growth rate in cold-acclimated bok choy.

In our study, CA plants exhibited high *BramMDH1* expression due to promoter demethylation. Moreover, *35S::BramMDH1* showed enhanced heat tolerance and a higher growth rate. Surprisingly, Aza-treated *B. rapa*, which also exhibits high *BramMDH1* expression, demonstrated no significant increase in heat tolerance compared with that of untreated *B. rapa* (Fig. 4D). Moreover, organic acids, which were elevated in CA and *35S::BramMDH1* plants, were not elevated in Aza-treated *B. rapa* (Fig. 4E). Aza is a specific inhibitor of DNA methylation (Goffin and Eisenhauer, 2002; Zhong *et al.*, 2013). In cold-acclimated *B. rapa*, however, some genes experienced increased methylation while some experienced reduced methylation. Therefore, we suggest that DNA methylation also plays an important role in increasing heat tolerance in CA plants. Likewise, in honeybee caste determination, both up- and down-methylation in the brains of workers and the queen have been detected (Lyko *et al.*, 2010), which cannot be explained by only the up-regulation of *Dnmts* in one of the castes. Together, our experimental and MeDIP-seq data support a model where enhanced heat tolerance and higher growth rate in CA plants are attributed to elevated organic acids and enhanced photosynthesis, respectively. These changes are associated with DNA methylation and demethylation during cold acclimation. Our findings may aid in developing a deeper understanding of cross-adaptation in plants.

## Supplementary Material

Supplementary Data
