# Supplementary material for: Cold acclimation alters DNA methylation patterns and confers tolerance to heat and increases growth rate in Brassica rapa
Source: J Exp Bot. 2017 Feb 1;68(5):1213–24. doi: 10.1093/jxb/erw496 (PMC5441862; doi:10.1093/jxb/erw496)
Supplement: Supplementary Data [file erw496_Supplementary_Data.zip › supplementary_figures_S1_S3_Tables_S1_S7.pdf]

Full title: Cold acclimation alters DNA methylation patterns in *Brassica rapa* and confers tolerance to heat and high growth rate

Running title: Cold acclimation and heat tolerance

Tongkun Liu, Ying Li, Weike Duan, Feiyi Huang, Xilin Hou*

Department of Horticulture, Nanjing Agricultural University, Nanjing, China,

Supplementary data

Figure S1. Chromosome distribution of reads in CK and CA leaves.

The x-axis indicates the number of windows, and the y-axis indicates the normalized read count of each window.

Figure S2. Distribution of reads and methylation peaks in CA and CK samples.

Density of reads across the genome in CA (**A**) and CK (**B**) samples. The x-axis indicates the number of CpGs in each 1000-bp window, and the y-axis indicates the proportion of reads in a specific range. The number of CpGs in 1000 bp reflects the CpG density of a specific region. Density of methylation peaks across the genome in CA (**C**) and CK (**D**) samples. Most reads and peaks tended to map to regions with a low number of CpGs. Distribution of reads among repetitive elements in CA (**E**) and CK (**F**) samples. The x-axis indicates repetitive elements, and the y-axis indicates the proportion of reads in a specific repetitive element.

Figure S3. mRNA expression levels of *BramMDH1* in wild-type (WT) and *35S::BramMDH1 Arabidopsis*.

Table S1. Primers used in this study.

Table S2. Methylation peak information.

Table S3. Numbers of differentially methylated regions among different gene regions.

Table S4. Top 10 significant GO groups involving up-methylated differentially methylated genes in CA plants. C, cellular component; F, molecular function; P, biological process.

Table S5. Top 10 significant GO groups involving down-methylated differentially methylated genes in CA plants. C, cellular component; F, molecular function; P, biological process.

Table S6. Top 10 significant KEGG pathways involving up-methylated differentially methylated genes in CA plants.

Table S7. Top 10 significant KEGG pathways involving down-methylated differentially methylated genes in CA plants.

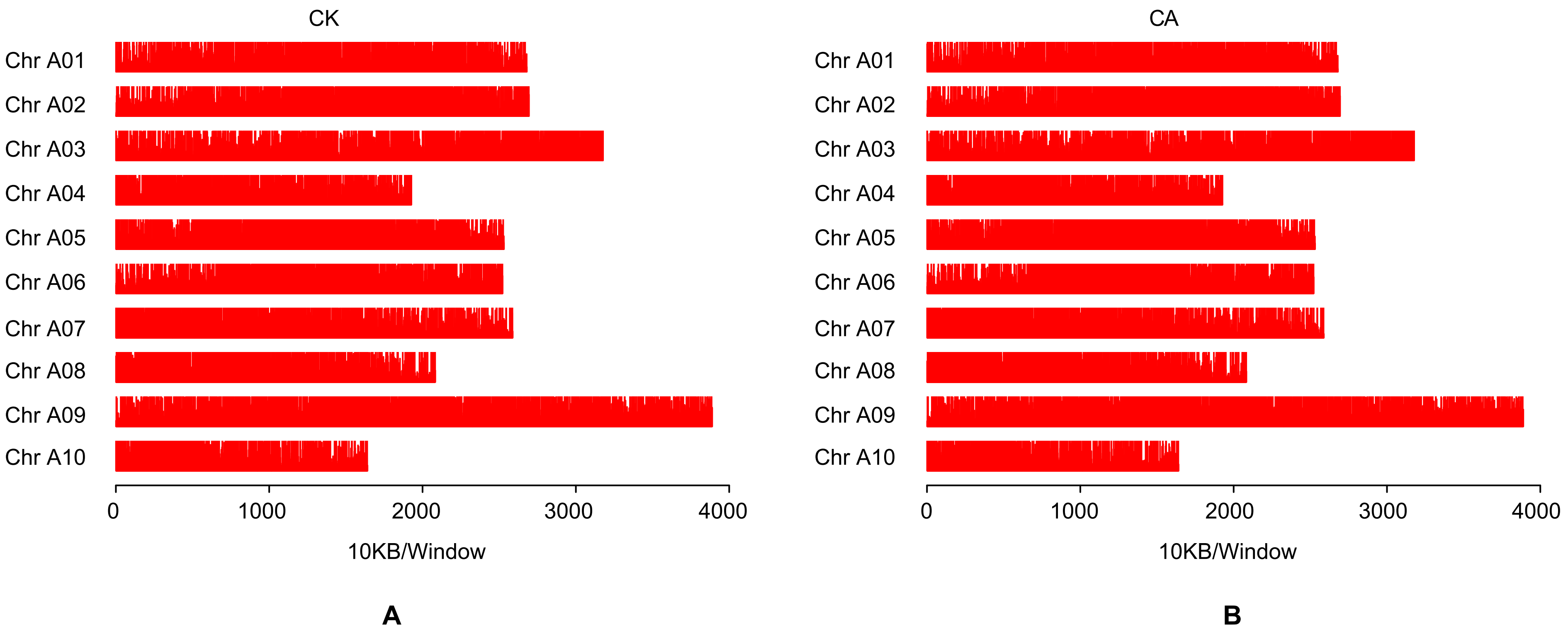

Figure S1. Chromosome distribution of reads in CK and CA leaves.

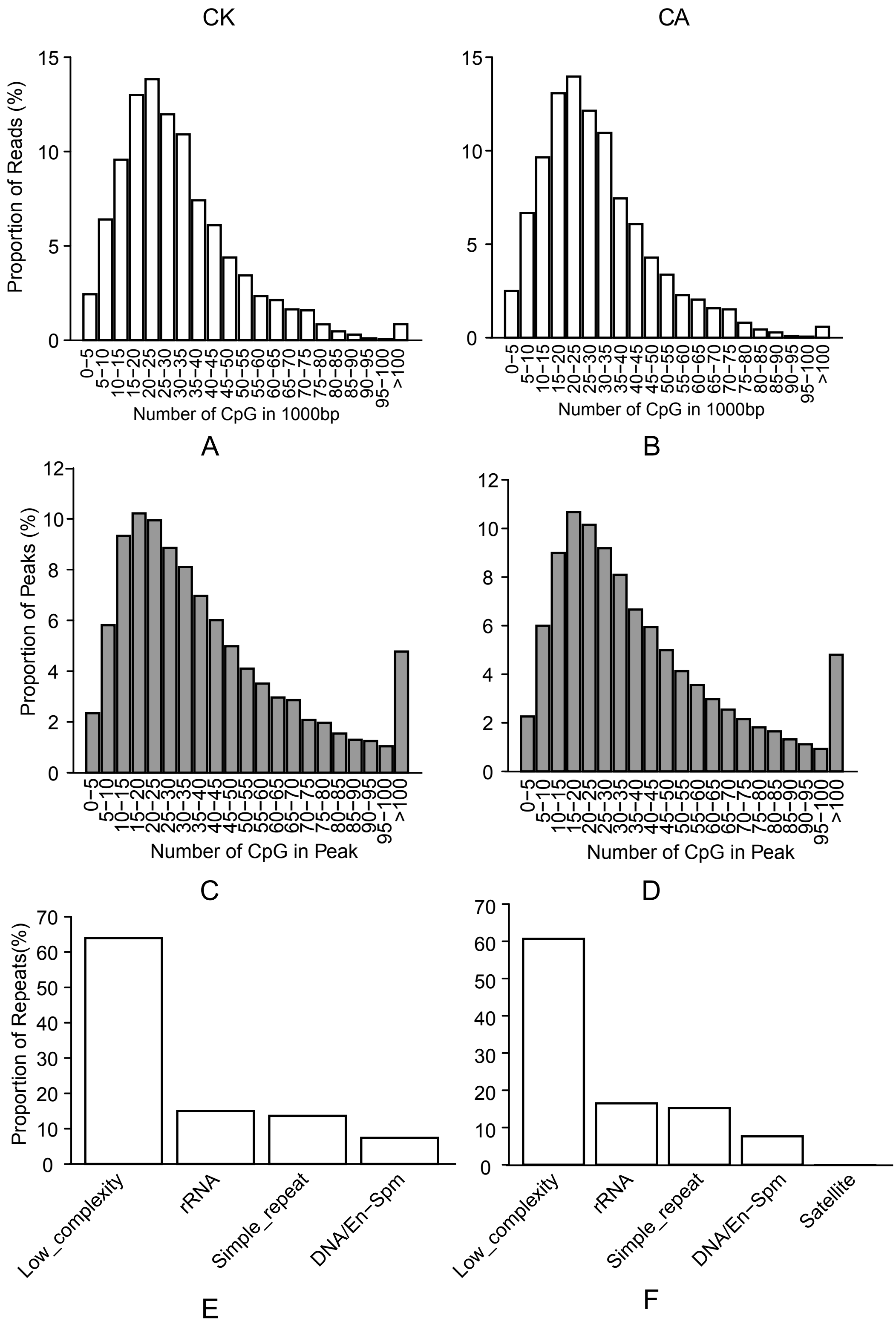

Figure S2. Distribution of reads and methylation peaks in CA and CK samples.

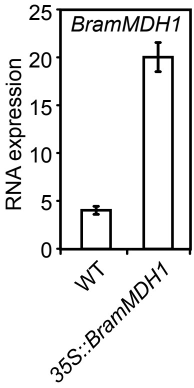

Figure S3. mRNA expression levels of *BramMDH1* in wild-type (WT) and *35S::BramMDH1* Arabidopsis.

Supplementary Table S1. Primers used in this study.

ID	Gene	Primer sequence	Function
Bra031307	*BraHYR1*	F: CTTGTGCACACCTGTTGTTA	BSP
		R: AAAACAAATATAAATAAAAC	
Bra039662	*BramMDH1*	F1: GAATATGACATGAGCGAAAT	BSP
		R1: ACCTCTATACCAAAATACCC	
		F2: GGGCCAAGACTTTCTACGCT	qPCR
		R2: GGTGAGAGCGGTGAGTACAT	
		F3:TGTTAAATTAAACAATAATA	promoter
		R3: TTTGGAGATGGAAGCTAGGGTTTAC	
		F4: CACCATGTACCCATACGATGTTCCAGATTACGCTTTCAGATCCGTCATCGT	HA-cDNA
		R4: TCATTGGTTGGCAAACTTAACA	
Bra020062	*BraTUA5*	F: CCGGAGCAGCTTATCTCTGG	qPCR
		R: AGCTTCCTCACACGGTCAAG	
Bra005441	*Brasec61*	F: GCCATTCTTGGCTTTTCTTCCAG	qPCR
		R: ATCGGCACCCGTAGTTGAAT	
Bra030911	*BraERMO3*	F: AGCTTCCCTCCCTACGGAAT	qPCR
		R: CGGAGGGATGTGAGGAACAC	
Bra014232	*BraTUA2*	F: ATCAAGACCAAGCGCACGA	qPCR
		R: CCCTCACCCACGTACCAGTG	
Bra035433	*BraADL3*	F: CCACAGTCTGGTGGGTCATC	qPCR
		R: GTGCAGGGTCCACTAAGACC	
Bra005522	*BraKAT2*	F1: TGTCGGTACTGTTCTGGCAC	qPCR
		R1: TCACGGTTCTAACCGGAACG	
		F2: AGAAAGGTATAGTATTGTTTAAAAA	BSP
		R2: AAAAAATTAATAATAAACTAAAAAAAA	
		F3: GATTATTAGTTTCGTTATTTTTTAAT	promoter
		R3: CCTGACCAAAGGATTTGTTG	
		F4: CACCATGTACCCATACGATGTTCCAGATTACGCTGAGAAAGCAATCGAGAGAC	HA-cDNA
		R4: CTAACGAGCGTCCTTGGACA	
Bra032749	*BraSHM4*	F1: TGGGCCTCGATCTACCTTCA	qPCR
		R1: AACGAGGCCACTGATGTGAG	
		F2: TTAGAAAAGTTTAAAGGAGAGTAGTTAGTA	BSP
		R2:AAAATCTAAAAACAAAAACTTTAAC	
		F3: GAAGATTAGATAGTAGTTATTAAGAGGGAG	
		R3:AAAATTATTTTTAAAAATAAAAAAAATC	
		F4: CCAAGAACAGAACAAAGCAGAC	promoter
		R4: CTCGGAGAATATGGTCGAGG	

		F5: CACCATGTACCCATACGATGTTCCAGATTACGCTGATCCAGTCTCAGCCT	HA-cDNA
		R5: CTAATCCTTGTACTTCATCGCA	
Bra031266	*Bra4CL2*	F1: CCCGGCGGAGATCTCTAAAC	qPCR
		R1: CACGACGTCTTCAGGGGAAA	
		F2: AGAAAAAGGTTAAAGATTATTTGTT	BSP
		R2: TAATTTACTACTCACTTAAAATACATATCA	
		F3: AAAATTTGATATGTATTTTAAGTGAGTAGT	
		R3: TATTATAACCCTAATAACACTCCCC	
		F4: TACTTGACGAAATGTCTGACATAAT	promoter
		R4: GATCACAAGTTCTTAAAACT	
		F5: CACCATGTACCCATACGATGTTCCAGATTACGCTTCCACACGAGAAGAGAC	HA-cDNA
		R5: TTACGTCAATCCATTAGCTA	
Bra000557	*BraHSFA2*	F: CTTCGTCTGTCGGTTCGTCA	qPCR
		R: CTTTGTCAGAAACGGCGGTG	
Bra006027	*BraHSP70*	F: CGTTCATCACAGCTGATGCG	qPCR
		R:CAGTCTGCACCTTAGGGACC	
Bra015922	*BraHSP101*	F: TTGCTTTACAGGAGGCGGAG	qPCR
		R: CACAGGAATCCCAGTCCACC	
Bra016729	*BraGAPDH*	F: AGAGCCGCTTCCTTCAACATCATT	reference gene
		R: TGGGCACACGGAAGGACATACC	

Note: The underlined sequence is HA tag.

Supplementary Table S2. Methylation peak information.

Sample	Total Peaks	Peak Mean Length	Peak Median Length	Peak Total Length	Peak Covered Size In Genome(%)
**CK**	19,001	1,526.91	1,322	29,012,891	10.19
**CA**	19,589	1,522	1,305	29,814,390	10.47

Supplementary Table S3. Numbers of differentially methylated regions among different gene regions.

Sample	upstream2k	CDS	Intron	downstream2k
**CK**	6,014	2,359	1,865	4,480
**CA**	6,074	2,373	1,901	4,576

Supplementary Table S4. Top 10 significant GO groups involving up-methylated differentially methylated genes in CA plants. C, cellular component; F, molecular function; P, biological process.

Terms for CDS.bed_Up_Gene_C

**Gene Ontology term**	**Cluster frequency**	**Genome frequency of use**	**Corrected P-value**	**Genes annotated to the term**
Golgi apparatus part	3 out of 86 genes, 3.5%	253 out of 33485 genes, 0.8%	1	Bra005835, Bra033434, Bra039898
Golgi membrane	2 out of 86 genes, 2.3%	137 out of 33485 genes, 0.4%	1	Bra005835, Bra033434
proteasome complex	2 out of 86 genes, 2.3%	138 out of 33485 genes, 0.4%	1	Bra021176, Bra022650
cytoplasm	59 out of 86 genes, 68.6%	20020 out of 33485 genes, 59.8%	1	Bra023737, Bra029934, Bra032052, Bra039379, Bra003673, Bra002992, Bra019526, Bra020195, Bra014088, Bra032698, Bra014126, Bra040348, Bra028131, Bra021176, Bra033434, Bra039898, Bra033805, Bra040811, Bra005331, Bra021487, Bra005835, Bra027169, Bra035662, Bra040444, Bra031086, Bra030447, Bra021511, Bra021508, Bra013231, Bra039217, Bra014837, Bra031288, Bra004758, Bra038727, Bra028321, Bra010199, Bra014264, Bra004819, Bra014162, Bra007767, Bra019279, Bra018130, Bra021642, Bra028581, Bra033335, Bra005394, Bra023137, Bra016005, Bra021213, Bra036994, Bra040853, Bra025250, Bra038702, Bra033856, Bra038667, Bra022650, Bra021754, Bra029317, Bra035691
endomembrane system	4 out of 86 genes, 4.7%	718 out of 33485 genes, 2.1%	1	Bra005835, Bra033434, Bra039379, Bra028581

extracellular region	15 out of 86 genes, 17.4%	4284 out of 33485 genes, 12.8%	1	Bra021508, Bra025736, Bra039217, Bra033434, Bra039898, Bra003673, Bra021579, Bra035548, Bra016005, Bra038727, Bra028321, Bra004891, Bra038667, Bra010199, Bra004819
intrinsic to plasma membrane	2 out of 86 genes, 2.3%	293 out of 33485 genes, 0.9%	1	Bra023940, Bra039898
cytoplasmic part	48 out of 86 genes, 55.8%	17135 out of 33485 genes, 51.2%	1	Bra023737, Bra029934, Bra032052, Bra039379, Bra003673, Bra002992, Bra019526, Bra020195, Bra014088, Bra032698, Bra014126, Bra040348, Bra028131, Bra021176, Bra033434, Bra033805, Bra039898, Bra040811, Bra021487, Bra005835, Bra035662, Bra040444, Bra031086, Bra030447, Bra021511, Bra013231, Bra021508, Bra039217, Bra014837, Bra004758, Bra038727, Bra028321, Bra010199, Bra004819, Bra014162, Bra007767, Bra019279, Bra018130, Bra021642, Bra028581, Bra005394, Bra016005, Bra021213, Bra036994, Bra040853, Bra025250, Bra038667, Bra029317
protein complex	9 out of 86 genes, 10.5%	2622 out of 33485 genes, 7.8%	1	Bra019279, Bra021176, Bra039379, Bra039898, Bra005835, Bra019531, Bra021511, Bra022650, Bra029317
plasmodesma	9 out of 86 genes, 10.5%	2691 out of 33485 genes, 8.0%	1	Bra039217, Bra020298, Bra021579, Bra021487, Bra005835, Bra040853, Bra033856, Bra003858, Bra010199

Terms for CDS.bed_Up_Gene_F

**Gene Ontology term**	**Cluster frequency**	**Genome frequency of use**	**Corrected P-value**	**Genes annotated to the term**
sulfurtransferase activity	2 out of 67 genes, 3.0%	20 out of 25348 genes, 0.1%	0.12807	Bra030447, Bra004758
guanylate cyclase	2 out of 67	22 out of 25348	0.15518	Bra025736, Bra003858

activity	genes, 3.0%	genes, 0.1%		
phosphorus-oxygen lyase activity	2 out of 67 genes, 3.0%	25 out of 25348 genes, 0.1%	0.20050	Bra025736, Bra003858
peptide receptor activity	2 out of 67 genes, 3.0%	28 out of 25348 genes, 0.1%	0.25135	Bra021176, Bra003858
signal transducer activity	6 out of 67 genes, 9.0%	649 out of 25348 genes, 2.6%	0.73998	Bra027169, Bra033856, Bra023940, Bra021176, Bra003858, Bra028581
molecular transducer activity	6 out of 67 genes, 9.0%	649 out of 25348 genes, 2.6%	0.73998	Bra027169, Bra033856, Bra023940, Bra021176, Bra003858, Bra028581
cyclase activity	2 out of 67 genes, 3.0%	55 out of 25348 genes, 0.2%	0.94319	Bra025736, Bra003858
transferase activity, transferring sulfur-containing groups	2 out of 67 genes, 3.0%	77 out of 25348 genes, 0.3%	1	Bra030447, Bra004758
peptide binding	2 out of 67 genes, 3.0%	77 out of 25348 genes, 0.3%	1	Bra003858, Bra039898
amide binding	2 out of 67 genes, 3.0%	98 out of 25348 genes, 0.4%	1	Bra003858, Bra039898

Terms for CDS.bed_Up_Gene_P

**Gene Ontology term**	**Cluster frequency**	**Genome frequency of use**	**Corrected P-value**	**Genes annotated to the term**
galactolipid biosynthetic process	5 out of 78 genes, 6.4%	243 out of 28587 genes, 0.9%	0.30171	Bra021487, Bra021213, Bra020088, Bra021642, Bra018609

galactolipid metabolic process	5 out of 78 genes, 6.4%	247 out of 28587 genes, 0.9%	0.32484	Bra021487, Bra021213, Bra020088, Bra021642, Bra018609
glycolipid biosynthetic process	5 out of 78 genes, 6.4%	271 out of 28587 genes, 0.9%	0.49271	Bra021487, Bra021213, Bra020088, Bra021642, Bra018609
glycolipid metabolic process	5 out of 78 genes, 6.4%	292 out of 28587 genes, 1.0%	0.68642	Bra021487, Bra021213, Bra020088, Bra021642, Bra018609
membrane lipid biosynthetic process	5 out of 78 genes, 6.4%	336 out of 28587 genes, 1.2%	1	Bra021487, Bra021213, Bra020088, Bra021642, Bra018609
response to wounding	9 out of 78 genes, 11.5%	1100 out of 28587 genes, 3.8%	1	Bra021508, Bra039217, Bra021176, Bra021487, Bra038727, Bra020195, Bra033856, Bra003858, Bra003778
membrane lipid metabolic process	5 out of 78 genes, 6.4%	395 out of 28587 genes, 1.4%	1	Bra021487, Bra021213, Bra020088, Bra021642, Bra018609
regulation of circadian rhythm	3 out of 78 genes, 3.8%	126 out of 28587 genes, 0.4%	1	Bra020195, Bra033856, Bra010199
response to fungus	11 out of 78 genes, 14.1%	1671 out of 28587 genes, 5.8%	1	Bra025736, Bra039217, Bra040061, Bra028581, Bra038727, Bra023940, Bra038667, Bra035477, Bra010199, Bra003778, Bra029317
response to DNA damage stimulus	6 out of 78 genes, 7.7%	603 out of 28587 genes, 2.1%	1	Bra019279, Bra021213, Bra020195, Bra021176, Bra032088, Bra035691

Terms for downstream2k.bed_Up_Gene_C

**Gene Ontology term**	**Cluster frequency**	**Genome frequency of use**	**Corrected P-value**	**Genes annotated to the term**
6-phosphofructokinase complex	2 out of 200 genes, 1.0%	17 out of 33485 genes, 0.1%	0.45507	Bra016799, Bra038519

extracellular region	35 out of 200 genes, 17.5%	4284 out of 33485 genes, 12.8%	1	Bra020126, Bra038935, Bra016004, Bra028132, Bra022141, Bra035106, Bra014089, Bra032681, Bra014200, Bra004892, Bra019790, Bra033462, Bra032689, Bra008806, Bra032679, Bra021508, Bra004915, Bra032711, Bra031616, Bra035366, Bra038727, Bra014180, Bra028321, Bra020299, Bra037739, Bra029898, Bra005168, Bra011290, Bra024975, Bra018132, Bra035392, Bra014154, Bra014836, Bra028044, Bra039189
cytosolic part	8 out of 200 genes, 4.0%	723 out of 33485 genes, 2.2%	1	Bra014558, Bra020724, Bra038519, Bra035392, Bra006486, Bra028132, Bra016799, Bra007707
membrane	79 out of 200 genes, 39.5%	11755 out of 33485 genes, 35.1%	1	Bra033476, Bra011004, Bra037545, Bra038935, Bra035106, Bra014188, Bra002991, Bra024483, Bra032746, Bra021798, Bra004892, Bra007633, Bra010966, Bra005330, Bra019790, Bra032672, Bra033254, Bra034257, Bra035669, Bra036037, Bra036748, Bra038460, Bra015765, Bra021508, Bra020724, Bra032711, Bra031288, Bra016799, Bra038727, Bra033868, Bra020299, Bra003858, Bra033252, Bra035386, Bra014558, Bra019652, Bra032683, Bra035398, Bra015633, Bra019767, Bra018132, Bra030830, Bra014179, Bra020412, Bra031287, Bra017946, Bra021173, Bra020411, Bra028132, Bra006486, Bra021758, Bra017945, Bra020037, Bra014231, Bra026481, Bra031722, Bra033462, Bra016069, Bra037254, Bra020994, Bra004915, Bra002096, Bra014189, Bra028321, Bra016161, Bra005168, Bra029894, Bra031307, Bra032741, Bra033075, Bra022517, Bra035392, Bra003584, Bra010773, Bra035394, Bra014836, Bra033474, Bra039189, Bra007707
cell periphery	58 out of 200 genes, 29.0%	8608 out of 33485 genes, 25.7%	1	Bra033476, Bra011004, Bra037545, Bra038935, Bra017946, Bra021173, Bra028132, Bra022141, Bra035106, Bra021758, Bra014188, Bra002991, Bra024483, Bra032746, Bra004892, Bra007633, Bra010966, Bra026481, Bra030432, Bra031722, Bra016069, Bra033254, Bra034257, Bra035669, Bra036037, Bra020994, Bra038460, Bra015765, Bra021508, Bra008806,

				Bra004915, Bra020724, Bra002096, Bra032711, Bra031288, Bra016799, Bra014189, Bra028321, Bra020299, Bra003858, Bra033252, Bra037739, Bra005168, Bra035386, Bra032683, Bra029894, Bra032741, Bra015633, Bra019767, Bra022517, Bra018132, Bra035394, Bra014179, Bra030830, Bra033474, Bra020412, Bra039189, Bra007707
plasma membrane	51 out of 200 genes, 25.5%	7499 out of 33485 genes, 22.4%	1	Bra033476, Bra011004, Bra037545, Bra038935, Bra017946, Bra021173, Bra028132, Bra035106, Bra021758, Bra014188, Bra002991, Bra024483, Bra032746, Bra007633, Bra010966, Bra026481, Bra031722, Bra016069, Bra033254, Bra034257, Bra035669, Bra036037, Bra020994, Bra038460, Bra021508, Bra004915, Bra020724, Bra002096, Bra032711, Bra031288, Bra014189, Bra028321, Bra020299, Bra003858, Bra033252, Bra005168, Bra035386, Bra032683, Bra029894, Bra032741, Bra015633, Bra019767, Bra022517, Bra018132, Bra035394, Bra014179, Bra030830, Bra033474, Bra020412, Bra039189, Bra007707
heterotrimeric G-protein complex	2 out of 200 genes, 1.0%	126 out of 33485 genes, 0.4%	1	Bra014189, Bra037545
microtubule associated complex	2 out of 200 genes, 1.0%	127 out of 33485 genes, 0.4%	1	Bra011415, Bra017946
extrinsic to internal side of plasma membrane	2 out of 200 genes, 1.0%	128 out of 33485 genes, 0.4%	1	Bra014189, Bra037545
extrinsic to plasma membrane	2 out of 200 genes, 1.0%	131 out of 33485 genes, 0.4%	1	Bra014189, Bra037545

Terms for downstream2k.bed_Up_Gene_F

Gene Ontology term	Cluster frequency	Genome frequency of use	Corrected P-value	Genes annotated to the term
abscisic acid glucosyltransferase activity	4 out of 159 genes, 2.5%	38 out of 25348 genes, 0.1%	0.01649	Bra032179, Bra011495, Bra011492, Bra031307
peptide receptor activity	3 out of 159 genes, 1.9%	28 out of 25348 genes, 0.1%	0.12514	Bra016069, Bra014231, Bra003858
flavonol 3-O-glucosyltransferase activity	2 out of 159 genes, 1.3%	12 out of 25348 genes, 0.0%	0.43829	Bra011495, Bra011492
glutamate-ammonia ligase activity	2 out of 159 genes, 1.3%	13 out of 25348 genes, 0.1%	0.51586	Bra035392, Bra028132
ammonia ligase activity	2 out of 159 genes, 1.3%	15 out of 25348 genes, 0.1%	0.68873	Bra035392, Bra028132
acid-ammonia (or amide) ligase activity	2 out of 159 genes, 1.3%	15 out of 25348 genes, 0.1%	0.68873	Bra035392, Bra028132
6-phosphofructokinase activity	2 out of 159 genes, 1.3%	17 out of 25348 genes, 0.1%	0.88477	Bra016799, Bra038519
quercetin 7-O-glucosyltransferase activity	3 out of 159 genes, 1.9%	55 out of 25348 genes, 0.2%	0.88571	Bra032179, Bra011495, Bra011492
phosphofructokinase activity	2 out of 159 genes, 1.3%	19 out of 25348 genes, 0.1%	1	Bra016799, Bra038519
signal transducer activity	10 out of 159 genes, 6.3%	649 out of 25348 genes, 2.6%	1	Bra029894, Bra014231, Bra018132, Bra028581, Bra016069, Bra033856, Bra003858, Bra033474, Bra019524, Bra036037

Terms for downstream2k.bed_Up_Gene_P

Gene Ontology term	Cluster frequency	Genome frequency of use	Corrected P-value	Genes annotated to the term
positive regulation of secondary metabolite biosynthetic process	9 out of 190 genes, 4.7%	349 out of 28587 genes, 1.2%	0.52423	Bra021173, Bra027771, Bra031516, Bra014154, Bra035394, Bra032158, Bra033856, Bra019524, Bra019645
regulation of lignin biosynthetic process	2 out of 190 genes, 1.1%	9 out of 28587 genes, 0.0%	1	Bra027771, Bra021173
regulation of secondary metabolic process	10 out of 190 genes, 5.3%	497 out of 28587 genes, 1.7%	1	Bra021173, Bra027771, Bra031516, Bra035394, Bra014154, Bra032158, Bra033856, Bra019524, Bra019645, Bra014323
positive regulation of flavonoid biosynthetic process	8 out of 190 genes, 4.2%	341 out of 28587 genes, 1.2%	1	Bra021173, Bra031516, Bra014154, Bra035394, Bra032158, Bra033856, Bra019524, Bra019645
regulation of secondary metabolite biosynthetic process	9 out of 190 genes, 4.7%	430 out of 28587 genes, 1.5%	1	Bra021173, Bra027771, Bra031516, Bra014154, Bra035394, Bra032158, Bra033856, Bra019524, Bra019645
response to wounding	16 out of 190 genes, 8.4%	1100 out of 28587 genes, 3.8%	1	Bra021508, Bra008806, Bra020724, Bra032711, Bra021173, Bra014306, Bra003659, Bra035106, Bra038727, Bra003858, Bra031307, Bra016069, Bra035394, Bra033856, Bra015634, Bra039855
glutamine biosynthetic process	2 out of 190 genes, 1.1%	13 out of 28587 genes, 0.0%	1	Bra035392, Bra028132
regulation of phenylpropanoid metabolic process	9 out of 190 genes, 4.7%	452 out of 28587 genes, 1.6%	1	Bra021173, Bra027771, Bra031516, Bra014154, Bra035394, Bra032158, Bra033856, Bra019524, Bra019645
regulation of cell death	18 out of 190	1377 out of	1	Bra020724, Bra021173, Bra033430, Bra038511, Bra035106, Bra032158,

	genes, 9.5%	28587 genes, 4.8%		Bra021758, Bra033431, Bra029898, Bra005168, Bra019645, Bra032746, Bra032741, Bra031516, Bra035394, Bra014154, Bra026187, Bra019524
glutamine family amino acid biosynthetic process	3 out of 190 genes, 1.6%	52 out of 28587 genes, 0.2%	1	Bra035392, Bra014323, Bra028132

Terms for Intron.bed_Up_Gene_C

Gene Ontology term	Cluster frequency	Genome frequency of use	Corrected P-value	Genes annotated to the term
Golgi-associated vesicle membrane	2 out of 66 genes, 3.0%	41 out of 33485 genes, 0.1%	0.18511	Bra005835, Bra011941
Golgi-associated vesicle	2 out of 66 genes, 3.0%	72 out of 33485 genes, 0.2%	0.55480	Bra005835, Bra011941
coated vesicle membrane	2 out of 66 genes, 3.0%	81 out of 33485 genes, 0.2%	0.69533	Bra005835, Bra011941
cytoplasmic membrane-bounded vesicle	5 out of 66 genes, 7.6%	710 out of 33485 genes, 2.1%	0.80918	Bra005835, Bra004819, Bra011941, Bra003673, Bra004890
cytoplasmic vesicle	5 out of 66 genes, 7.6%	710 out of 33485 genes, 2.1%	0.80918	Bra005835, Bra004819, Bra011941, Bra003673, Bra004890
membrane-bounded vesicle	5 out of 66 genes, 7.6%	725 out of 33485 genes, 2.2%	0.87871	Bra005835, Bra004819, Bra011941, Bra003673, Bra004890
vesicle	5 out of 66 genes, 7.6%	733 out of 33485 genes, 2.2%	0.91740	Bra005835, Bra004819, Bra011941, Bra003673, Bra004890
cytoplasmic vesicle membrane	2 out of 66 genes, 3.0%	95 out of 33485 genes, 0.3%	0.94148	Bra005835, Bra011941
cytoplasmic vesicle part	2 out of 66 genes, 3.0%	95 out of 33485 genes, 0.3%	0.94148	Bra005835, Bra011941

vesicle membrane	2 out of 66 genes, 3.0%	101 out of 33485 genes, 0.3%	1	Bra005835, Bra011941

Terms for Intron.bed_Up_Gene_F

Gene Ontology term	Cluster frequency	Genome frequency of use	Corrected P-value	Genes annotated to the term
cyclase activity	2 out of 56 genes, 3.6%	55 out of 25348 genes, 0.2%	0.54154	Bra025736, Bra040920
lyase activity	5 out of 56 genes, 8.9%	711 out of 25348 genes, 2.8%	1	Bra021487, Bra025736, Bra040809, Bra035662, Bra040920
transcription factor binding transcription factor activity	2 out of 56 genes, 3.6%	101 out of 25348 genes, 0.4%	1	Bra026030, Bra028356
transcription cofactor activity	2 out of 56 genes, 3.6%	101 out of 25348 genes, 0.4%	1	Bra026030, Bra028356
protein binding transcription factor activity	2 out of 56 genes, 3.6%	112 out of 25348 genes, 0.4%	1	Bra026030, Bra028356
transferase activity	19 out of 56 genes, 33.9%	5686 out of 25348 genes, 22.4%	1	Bra029355, Bra014837, Bra031288, Bra007883, Bra032743, Bra014264, Bra025736, Bra018213, Bra020298, Bra030961, Bra021579, Bra013661, Bra021487, Bra026030, Bra031343, Bra027169, Bra035662, Bra030447, Bra014323
transferase activity, transferring	4 out of 56	639 out of	1	Bra026030, Bra032743, Bra029355, Bra014323

acyl groups	genes, 7.1%	25348 genes, 2.5%		
unfolded protein binding	2 out of 56 genes, 3.6%	196 out of 25348 genes, 0.8%	1	Bra038702, Bra039379
catalytic activity	38 out of 56 genes, 67.9%	14523 out of 25348 genes, 57.3%	1	Bra003903, Bra007883, Bra002992, Bra035548, Bra020195, Bra021492, Bra040920, Bra032698, Bra018213, Bra040811, Bra021579, Bra013661, Bra021487, Bra026030, Bra031343, Bra027169, Bra035662, Bra019636, Bra030447, Bra021511, Bra029355, Bra011941, Bra031288, Bra014837, Bra032743, Bra028321, Bra014264, Bra019279, Bra025736, Bra040809, Bra020298, Bra030961, Bra005394, Bra025250, Bra038667, Bra022650, Bra035691, Bra014323
oxidoreductase activity, acting on paired donors, with incorporation or reduction of molecular oxygen	4 out of 56 genes, 7.1%	756 out of 25348 genes, 3.0%	1	Bra038667, Bra021492, Bra019636, Bra005394

Terms for Intron.bed_Up_Gene_P

Gene Ontology term	Cluster frequency	Genome frequency of use	Corrected P-value	Genes annotated to the term
response to DNA damage stimulus	6 out of 63 genes, 9.5%	603 out of 28587 genes, 2.1%	0.96776	Bra019279, Bra020195, Bra003559, Bra021176, Bra032088, Bra035691
DNA repair	5 out of 63 genes, 7.9%	499 out of 28587 genes, 1.7%	1	Bra019279, Bra020195, Bra003559, Bra032088, Bra035691
plant-type cell wall modification involved in multidimensional cell growth	2 out of 63 genes, 3.2%	50 out of 28587 genes, 0.2%	1	Bra004891, Bra004890
cell wall modification involved in	2 out of 63 genes,	58 out of 28587	1	Bra004891, Bra004890

multidimensional cell growth	3.2%	genes, 0.2%		
plant-type cell wall loosening	2 out of 63 genes, 3.2%	60 out of 28587 genes, 0.2%	1	Bra004891, Bra004890
double-strand break repair	3 out of 63 genes, 4.8%	198 out of 28587 genes, 0.7%	1	Bra020195, Bra003559, Bra032088
response to wounding	7 out of 63 genes, 11.1%	1100 out of 28587 genes, 3.8%	1	Bra021487, Bra020195, Bra021492, Bra039217, Bra021176, Bra013661, Bra003778
endosperm development	2 out of 63 genes, 3.2%	79 out of 28587 genes, 0.3%	1	Bra018213, Bra020298
programmed cell death	3 out of 63 genes, 4.8%	226 out of 28587 genes, 0.8%	1	Bra019279, Bra021492, Bra013661
DNA recombination	4 out of 63 genes, 6.3%	469 out of 28587 genes, 1.6%	1	Bra020195, Bra003559, Bra032088, Bra035691

Terms for upstream2k.bed_Up_Gene_C

**Gene Ontology term**	**Cluster frequency**	**Genome frequency of use**	**Corrected P-value**	**Genes annotated to the term**
nuclear envelope	7 out of 264 genes, 2.7%	197 out of 33485 genes, 0.6%	0.12697	Bra015851, Bra039379, Bra014549, Bra023224, Bra007281, Bra027596, Bra033767
perinuclear region of cytoplasm	2 out of 264 genes, 0.8%	11 out of 33485 genes, 0.0%	0.40946	Bra021213, Bra023224
nucleoplasm	8 out of 264 genes, 3.0%	366 out of 33485 genes, 1.1%	1	Bra003602, Bra039797, Bra002036, Bra033767, Bra019819, Bra019531, Bra005172, Bra023224

protein complex	32 out of 264 genes, 12.1%	2622 out of 33485 genes, 7.8%	1	Bra020934, Bra037545, Bra039889, Bra017946, Bra039379, Bra039797, Bra010967, Bra033767, Bra014835, Bra030576, Bra039898, Bra031294, Bra013450, Bra038247, Bra032766, Bra014787, Bra021511, Bra025605, Bra021186, Bra030304, Bra003928, Bra008829, Bra019815, Bra031087, Bra015766, Bra014549, Bra023341, Bra014033, Bra031233, Bra014882, Bra019531, Bra029317
microtubule associated complex	4 out of 264 genes, 1.5%	127 out of 33485 genes, 0.4%	1	Bra021186, Bra017946, Bra030304, Bra014787
cytoskeletal part	8 out of 264 genes, 3.0%	419 out of 33485 genes, 1.3%	1	Bra013450, Bra019815, Bra021186, Bra017946, Bra039379, Bra030304, Bra014787, Bra030576
intrinsic to mitochondrial outer membrane	2 out of 264 genes, 0.8%	33 out of 33485 genes, 0.1%	1	Bra014254, Bra040813
integral to mitochondrial outer membrane	2 out of 264 genes, 0.8%	33 out of 33485 genes, 0.1%	1	Bra014254, Bra040813
endomembrane system	11 out of 264 genes, 4.2%	718 out of 33485 genes, 2.1%	1	Bra039379, Bra033434, Bra001775, Bra007281, Bra033767, Bra015851, Bra032766, Bra028583, Bra023224, Bra014549, Bra027596
microtubule	5 out of 264 genes, 1.9%	215 out of 33485 genes, 0.6%	1	Bra013450, Bra021186, Bra017946, Bra039379, Bra030304

Terms for upstream2k.bed_Up_Gene_F

Gene Ontology term	Cluster frequency	Genome frequency of use	Corrected P-value	Genes annotated to the term
polyamine transmembrane transporter activity	2 out of 203 genes, 1.0%	10 out of 25348 genes, 0.0%	0.47903	Bra038217, Bra027411
4 iron, 4 sulfur cluster binding	3 out of 203 genes, 1.5%	47 out of 25348 genes, 0.2%	1	Bra040756, Bra004758, Bra023341
galactosylxylosylprotein 3-beta-galactosyltransferase activity	2 out of 203 genes, 1.0%	17 out of 25348 genes, 0.1%	1	Bra010198, Bra018103
sulfurtransferase activity	2 out of 203 genes, 1.0%	20 out of 25348 genes, 0.1%	1	Bra030447, Bra004758
cationic amino acid transmembrane transporter activity	2 out of 203 genes, 1.0%	23 out of 25348 genes, 0.1%	1	Bra038217, Bra027411
carboxylic acid transmembrane transporter activity	5 out of 203 genes, 2.5%	183 out of 25348 genes, 0.7%	1	Bra038217, Bra014272, Bra027411, Bra014029, Bra020908
organic acid transmembrane transporter activity	5 out of 203 genes, 2.5%	191 out of 25348 genes, 0.8%	1	Bra038217, Bra014272, Bra027411, Bra014029, Bra020908
S-adenosylmethionine-dependent methyltransferase activity	5 out of 203 genes, 2.5%	199 out of 25348 genes, 0.8%	1	Bra028133, Bra011414, Bra030447, Bra019031, Bra019711
motor activity	4 out of 203 genes, 2.0%	135 out of 25348 genes, 0.5%	1	Bra021186, Bra017946, Bra030304, Bra030576
transferase activity, transferring sulfur-containing groups	3 out of 203 genes, 1.5%	77 out of 25348 genes, 0.3%	1	Bra017364, Bra030447, Bra004758

Terms for upstream2k.bed_Up_Gene_P

Gene Ontology term	Cluster frequency	Genome frequency of use	Corrected P-value	Genes annotated to the term
negative regulation of defense response	20 out of 236 genes, 8.5%	978 out of 28587 genes, 3.4%	0.20936	Bra005525, Bra014635, Bra020776, Bra003927, Bra020249, Bra014313, Bra032747, Bra019529, Bra003778, Bra032746, Bra032740, Bra005680, Bra005529, Bra031294, Bra035511, Bra035508, Bra017364, Bra023224, Bra004309, Bra035384
negative regulation of response to stimulus	23 out of 236 genes, 9.7%	1214 out of 28587 genes, 4.2%	0.21151	Bra005525, Bra014635, Bra020776, Bra003927, Bra020249, Bra014313, Bra032747, Bra019529, Bra008758, Bra003778, Bra032746, Bra030917, Bra032740, Bra005680, Bra005529, Bra031294, Bra035511, Bra014882, Bra035508, Bra017364, Bra023224, Bra004309, Bra035384
innate immune response	32 out of 236 genes, 13.6%	2130 out of 28587 genes, 7.5%	0.79962	Bra032160, Bra005525, Bra014635, Bra029311, Bra007430, Bra039797, Bra023009, Bra035249, Bra019819, Bra003927, Bra020249, Bra014313, Bra010199, Bra032747, Bra002018, Bra019529, Bra003778, Bra032746, Bra030917, Bra032740, Bra005680, Bra001690, Bra005529, Bra035511, Bra023940, Bra028583, Bra003585, Bra017364, Bra023224, Bra021511, Bra004309, Bra035384
immune response	32 out of 236 genes, 13.6%	2138 out of 28587 genes, 7.5%	0.85121	Bra032160, Bra005525, Bra014635, Bra029311, Bra007430, Bra039797, Bra023009, Bra035249, Bra019819, Bra003927, Bra020249, Bra014313, Bra010199, Bra032747, Bra002018, Bra019529, Bra003778, Bra032746, Bra030917, Bra032740, Bra005680, Bra001690, Bra005529, Bra035511, Bra023940, Bra028583, Bra003585, Bra017364, Bra023224, Bra021511, Bra004309, Bra035384
negative regulation of biological process	41 out of 236 genes, 17.4%	3111 out of 28587 genes, 10.9%	1	Bra035249, Bra033767, Bra003927, Bra020249, Bra024250, Bra014313, Bra035361, Bra019529, Bra032746, Bra030917, Bra005680, Bra005529, Bra031294, Bra035511, Bra013450, Bra038247, Bra016162, Bra003585, Bra035508, Bra023224, Bra004309, Bra014635, Bra005525, Bra020776,

				Bra019819, Bra020182, Bra032747, Bra010199, Bra008758, Bra021759, Bra003778, Bra032740, Bra035381, Bra001690, Bra005393, Bra021213, Bra014551, Bra014882, Bra006186, Bra017364, Bra035384
polyamine transport	2 out of 236 genes, 0.8%	8 out of 28587 genes, 0.0%	1	Bra038217, Bra027411
regulation of cell death	22 out of 236 genes, 9.3%	1377 out of 28587 genes, 4.8%	1	Bra005525, Bra014635, Bra020776, Bra035107, Bra003975, Bra003927, Bra014313, Bra032747, Bra021759, Bra003778, Bra019645, Bra032746, Bra032740, Bra005680, Bra005529, Bra035511, Bra023940, Bra006186, Bra017364, Bra023224, Bra004309, Bra035384
regulation of response to stress	27 out of 236 genes, 11.4%	1830 out of 28587 genes, 6.4%	1	Bra005525, Bra014635, Bra020776, Bra007430, Bra003975, Bra003927, Bra020249, Bra014313, Bra032747, Bra019529, Bra008758, Bra021759, Bra003778, Bra019645, Bra032746, Bra030917, Bra032740, Bra005680, Bra005529, Bra031294, Bra035511, Bra035508, Bra006186, Bra017364, Bra023224, Bra004309, Bra035384
systemic acquired resistance, salicylic acid mediated signaling pathway	16 out of 236 genes, 6.8%	876 out of 28587 genes, 3.1%	1	Bra005525, Bra014635, Bra032740, Bra005680, Bra005529, Bra035511, Bra003927, Bra014313, Bra032747, Bra017364, Bra023224, Bra019529, Bra004309, Bra035384, Bra003778, Bra032746
regulation of defense response	26 out of 236 genes, 11.0%	1778 out of 28587 genes, 6.2%	1	Bra005525, Bra014635, Bra020776, Bra007430, Bra003975, Bra003927, Bra020249, Bra014313, Bra032747, Bra019529, Bra021759, Bra003778, Bra019645, Bra032746, Bra030917, Bra032740, Bra005680, Bra005529, Bra031294, Bra035511, Bra035508, Bra006186, Bra017364, Bra023224, Bra004309, Bra035384

Supplementary Table S5. Top 10 significant GO groups involving down-methylated differentially methylated genes in CA plants. C, cellular component; F, molecular function; P, biological process.

Terms for CDS.bed_Down_Gene_C

**Gene Ontology term**	**Cluster frequency**	**Genome frequency of use**	**Corrected P-value**	**Genes annotated to the term**
extracellular region	17 out of 77 genes, 22.1%	4284 out of 33485 genes, 12.8%	1	Bra005549, Bra025640, Bra031074, Bra033453, Bra030913, Bra019504, Bra005534, Bra030911, Bra014095, Bra021868, Bra021913, Bra018663, Bra038254, Bra038610, Bra040998, Bra030511, Bra005517
stromule	2 out of 77 genes, 2.6%	115 out of 33485 genes, 0.3%	1	Bra038030, Bra019504
ribosome	5 out of 77 genes, 6.5%	905 out of 33485 genes, 2.7%	1	Bra022247, Bra030913, Bra031984, Bra030911, Bra024598
cytosolic ribosome	4 out of 77 genes, 5.2%	663 out of 33485 genes, 2.0%	1	Bra022247, Bra030913, Bra030911, Bra024598
chloroplast	23 out of 77 genes, 29.9%	7419 out of 33485 genes, 22.2%	1	Bra033249, Bra030913, Bra019504, Bra021173, Bra005534, Bra030911, Bra005879, Bra030565, Bra022247, Bra014095, Bra032746, Bra030250, Bra038030, Bra038262, Bra031984, Bra021913, Bra021868, Bra014108, Bra002967, Bra031306, Bra005563, Bra013999, Bra040998
cytosolic part	4 out of 77 genes, 5.2%	723 out of 33485 genes, 2.2%	1	Bra022247, Bra030913, Bra030911, Bra024598
plastid envelope	6 out of 77	1336 out of	1	Bra038030, Bra030913, Bra038262, Bra019504, Bra021173, Bra030911

	genes, 7.8%	33485 genes, 4.0%		
plant-type cell wall	4 out of 77 genes, 5.2%	742 out of 33485 genes, 2.2%	1	Bra025640, Bra030913, Bra030511, Bra021217
small ribosomal subunit	2 out of 77 genes, 2.6%	224 out of 33485 genes, 0.7%	1	Bra031984, Bra024598
ribonucleoprotein complex	5 out of 77 genes, 6.5%	1096 out of 33485 genes, 3.3%	1	Bra022247, Bra030913, Bra031984, Bra030911, Bra024598

Terms for CDS.bed_Down_Gene_F

**Gene Ontology term**	**Cluster frequency**	**Genome frequency of use**	**Corrected P-value**	**Genes annotated to the term**
transferase activity, transferring phosphorus-containing groups	17 out of 65 genes, 26.2%	2964 out of 25348 genes, 11.7%	0.08659	Bra035436, Bra027548, Bra027691, Bra005534, Bra027271, Bra003650, Bra032746, Bra018183, Bra031984, Bra021868, Bra021913, Bra027046, Bra004856, Bra005563, Bra013999, Bra014042, Bra040998
kinase activity	15 out of 65 genes, 23.1%	2617 out of 25348 genes, 10.3%	0.18469	Bra035436, Bra018183, Bra027548, Bra021913, Bra021868, Bra027691, Bra005534, Bra027271, Bra005563, Bra004856, Bra013999, Bra014042, Bra040998, Bra003650, Bra032746
catalytic activity	48 out of 65 genes, 73.8%	14523 out of 25348 genes, 57.3%	0.35980	Bra035436, Bra025640, Bra033453, Bra027548, Bra019504, Bra014826, Bra021173, Bra002392, Bra011900, Bra030911, Bra005879, Bra031263, Bra032746, Bra021217, Bra030250, Bra038030, Bra018183, Bra035675, Bra031984, Bra021913, Bra014405, Bra021868, Bra027046, Bra018663, Bra014108, Bra004856, Bra038254, Bra031306, Bra019081, Bra040998,

				Bra030511, Bra033249, Bra030913, Bra027691, Bra005534, Bra027271, Bra022247, Bra032731, Bra019382, Bra003650, Bra030455, Bra034939, Bra002967, Bra005563, Bra014620, Bra038610, Bra013999, Bra014042
transferase activity	24 out of 65 genes, 36.9%	5686 out of 25348 genes, 22.4%	0.48286	Bra035436, Bra033249, Bra027548, Bra027691, Bra005534, Bra011900, Bra027271, Bra005879, Bra003650, Bra032746, Bra038030, Bra018183, Bra035675, Bra031984, Bra021913, Bra021868, Bra027046, Bra005563, Bra031306, Bra004856, Bra019081, Bra013999, Bra014042, Bra040998
hydrolase activity, acting on ester bonds	10 out of 65 genes, 15.4%	1759 out of 25348 genes, 6.9%	1	Bra033249, Bra030913, Bra030455, Bra014405, Bra014826, Bra030911, Bra034939, Bra038610, Bra030511, Bra021217
phosphoric ester hydrolase activity	6 out of 65 genes, 9.2%	772 out of 25348 genes, 3.0%	1	Bra034939, Bra033249, Bra038610, Bra030455, Bra014405, Bra014826
phosphatase activity	5 out of 65 genes, 7.7%	605 out of 25348 genes, 2.4%	1	Bra033249, Bra038610, Bra030455, Bra014405, Bra014826
hydrolase activity	20 out of 65 genes, 30.8%	5105 out of 25348 genes, 20.1%	1	Bra035436, Bra025640, Bra033249, Bra033453, Bra030913, Bra014826, Bra021173, Bra030911, Bra032731, Bra021217, Bra030250, Bra038030, Bra031984, Bra030455, Bra014405, Bra014108, Bra034939, Bra038610, Bra014620, Bra030511
aspartyl esterase activity	2 out of 65 genes, 3.1%	106 out of 25348 genes, 0.4%	1	Bra030511, Bra021217
protein serine/threonine phosphatase activity	3 out of 65 genes, 4.6%	277 out of 25348 genes, 1.1%	1	Bra038610, Bra030455, Bra014405

Terms for CDS.bed_Down_Gene_P

**Gene Ontology term**	**Cluster frequency**	**Genome frequency of use**	**Corrected P-value**	**Genes annotated to the term**
response to organic cyclic compound	16 out of 70 genes, 22.9%	2490 out of 28587 genes, 8.7%	0.15161	Bra036754, Bra021173, Bra002392, Bra005534, Bra014411, Bra032746, Bra031984, Bra021868, Bra021913, Bra017883, Bra018663, Bra005563, Bra013999, Bra039392, Bra030511, Bra040998
S-glycoside catabolic process	3 out of 70 genes, 4.3%	70 out of 28587 genes, 0.2%	0.37637	Bra030913, Bra021173, Bra030911
glycosinolate catabolic process	3 out of 70 genes, 4.3%	70 out of 28587 genes, 0.2%	0.37637	Bra030913, Bra021173, Bra030911
glucosinolate catabolic process	3 out of 70 genes, 4.3%	70 out of 28587 genes, 0.2%	0.37637	Bra030913, Bra021173, Bra030911
regulation of cellular response to stress	10 out of 70 genes, 14.3%	1287 out of 28587 genes, 4.5%	0.61609	Bra027548, Bra031984, Bra021913, Bra021868, Bra021173, Bra005534, Bra018663, Bra005563, Bra031263, Bra032746
cellular response to organic cyclic compound	11 out of 70 genes, 15.7%	1527 out of 28587 genes, 5.3%	0.63393	Bra031984, Bra036754, Bra017883, Bra021913, Bra021868, Bra021173, Bra005534, Bra018663, Bra005563, Bra013999, Bra032746
primary metabolic process	52 out of 70 genes, 74.3%	16037 out of 28587 genes, 56.1%	0.69612	Bra035436, Bra025640, Bra033453, Bra027548, Bra019504, Bra032827, Bra014826, Bra021173, Bra036712, Bra002392, Bra011900, Bra030911, Bra005879, Bra031263, Bra014431, Bra032746, Bra038030, Bra018183, Bra021109, Bra035675, Bra031984, Bra014405, Bra027046, Bra018663, Bra024598, Bra014108, Bra031306, Bra004856, Bra038254, Bra019081, Bra039392, Bra040998, Bra030511, Bra033249, Bra031195, Bra030913, Bra036754, Bra036757, Bra027691, Bra027271, Bra022247, Bra019382, Bra003650, Bra030455, Bra017883, Bra034939, Bra005563, Bra002967,

				Bra039810, Bra013999, Bra014042, Bra033685
sulfur compound catabolic process	3 out of 70 genes, 4.3%	91 out of 28587 genes, 0.3%	0.80519	Bra030913, Bra021173, Bra030911
response to endoplasmic reticulum stress	9 out of 70 genes, 12.9%	1109 out of 28587 genes, 3.9%	0.82303	Bra021913, Bra021868, Bra027691, Bra005534, Bra018663, Bra022604, Bra019382, Bra040998, Bra032746
polyprenol metabolic process	2 out of 70 genes, 2.9%	26 out of 28587 genes, 0.1%	1	Bra031984, Bra027046

Terms for downstream2k.bed_Down_Gene_C

Gene Ontology term	Cluster frequency	Genome frequency of use	Corrected P-value	Genes annotated to the term
extrinsic to membrane	3 out of 152 genes, 2.0%	203 out of 33485 genes, 0.6%	1	Bra014107, Bra018119, Bra014136
plastoglobule	2 out of 152 genes, 1.3%	115 out of 33485 genes, 0.3%	1	Bra021912, Bra020970
integral to organelle membrane	2 out of 152 genes, 1.3%	137 out of 33485 genes, 0.4%	1	Bra033849, Bra006282
chromatin	2 out of 152 genes, 1.3%	142 out of 33485 genes, 0.4%	1	Bra039810, Bra006621
intracellular part	136 out of 152 genes,	28901 out of 33485 genes,	1	Bra021453, Bra030285, Bra031231, Bra040819, Bra032750, Bra035466, Bra014117, Bra014627, Bra018304, Bra028322, Bra030261, Bra004757,

	89.5%	86.3%		Bra010929, Bra033455, Bra014863, Bra014081, Bra036713, Bra018223, Bra014085, Bra030483, Bra028532, Bra009127, Bra031394, Bra040281, Bra019333, Bra038942, Bra014621, Bra019642, Bra014136, Bra032770, Bra021592, Bra041113, Bra030912, Bra033872, Bra031263, Bra026317, Bra014234, Bra014431, Bra034878, Bra027442, Bra006282, Bra003669, Bra039962, Bra009325, Bra033248, Bra031306, Bra022611, Bra038699, Bra014315, Bra035662, Bra014107, Bra021914, Bra032335, Bra014427, Bra021216, Bra005159, Bra014232, Bra025555, Bra022705, Bra033454, Bra003757, Bra021165, Bra003764, Bra040853, Bra021912, Bra034252, Bra038744, Bra014433, Bra014143, Bra018174, Bra039376, Bra005521, Bra003790, Bra014071, Bra005022, Bra005595, Bra016206, Bra039487, Bra014628, Bra033457, Bra030264, Bra029950, Bra037467, Bra003872, Bra018472, Bra013994, Bra004752, Bra036114, Bra030880, Bra014426, Bra039954, Bra031195, Bra014550, Bra022758, Bra014640, Bra028108, Bra016025, Bra033849, Bra030861, Bra031265, Bra027179, Bra038441, Bra032726, Bra039810, Bra014862, Bra006621, Bra021748, Bra039186, Bra038100, Bra038106, Bra016479, Bra021750, Bra007627, Bra036324, Bra038346, Bra014764, Bra021519, Bra014361, Bra019081, Bra021800, Bra036238, Bra021858, Bra018119, Bra016098, Bra031241, Bra013872, Bra020970, Bra004040, Bra032749, Bra035443, Bra013690, Bra031245, Bra021871, Bra018298, Bra036937, Bra033685
anchored to membrane	4 out of 152 genes, 2.6%	463 out of 33485 genes, 1.4%	1	Bra020151, Bra037422, Bra014427, Bra033248
intrinsic to organelle membrane	2 out of 152 genes, 1.3%	164 out of 33485 genes, 0.5%	1	Bra033849, Bra006282
intracellular	136 out of	29024 out of	1	Bra021453, Bra030285, Bra031231, Bra040819, Bra032750, Bra035466,

	152 genes, 89.5%	33485 genes, 86.7%		Bra014117, Bra014627, Bra018304, Bra028322, Bra030261, Bra004757, Bra010929, Bra033455, Bra014863, Bra014081, Bra036713, Bra018223, Bra014085, Bra030483, Bra028532, Bra009127, Bra031394, Bra040281, Bra019333, Bra038942, Bra014621, Bra019642, Bra014136, Bra032770, Bra021592, Bra041113, Bra030912, Bra033872, Bra031263, Bra026317, Bra014234, Bra014431, Bra034878, Bra027442, Bra006282, Bra003669, Bra039962, Bra009325, Bra033248, Bra031306, Bra022611, Bra038699, Bra014315, Bra035662, Bra014107, Bra021914, Bra032335, Bra014427, Bra021216, Bra005159, Bra014232, Bra025555, Bra022705, Bra033454, Bra003757, Bra021165, Bra003764, Bra040853, Bra021912, Bra034252, Bra038744, Bra014433, Bra014143, Bra018174, Bra039376, Bra005521, Bra003790, Bra014071, Bra005022, Bra005595, Bra016206, Bra039487, Bra014628, Bra033457, Bra030264, Bra029950, Bra037467, Bra003872, Bra018472, Bra013994, Bra004752, Bra036114, Bra030880, Bra014426, Bra039954, Bra031195, Bra014550, Bra022758, Bra014640, Bra028108, Bra016025, Bra033849, Bra030861, Bra031265, Bra027179, Bra038441, Bra032726, Bra039810, Bra014862, Bra006621, Bra021748, Bra039186, Bra038100, Bra038106, Bra016479, Bra021750, Bra007627, Bra036324, Bra038346, Bra014764, Bra021519, Bra014361, Bra019081, Bra021800, Bra036238, Bra021858, Bra018119, Bra016098, Bra031241, Bra013872, Bra020970, Bra004040, Bra032749, Bra035443, Bra013690, Bra031245, Bra021871, Bra018298, Bra036937, Bra033685
chromosome	3 out of 152 genes, 2.0%	330 out of 33485 genes, 1.0%	1	Bra039810, Bra021800, Bra006621
chloroplast membrane	2 out of 152 genes, 1.3%	178 out of 33485 genes, 0.5%	1	Bra013690, Bra006282

Terms for downstream2k.bed_Down_Gene_F

Gene Ontology term	Cluster frequency	Genome frequency of use	Corrected P-value	Genes annotated to the term
4-coumarate-CoA ligase activity	2 out of 113 genes, 1.8%	22 out of 25348 genes, 0.1%	0.59238	Bra031265, Bra031263
inorganic diphosphatase activity	2 out of 113 genes, 1.8%	34 out of 25348 genes, 0.1%	1	Bra016025, Bra040819
CoA-ligase activity	2 out of 113 genes, 1.8%	56 out of 25348 genes, 0.2%	1	Bra031265, Bra031263
acid-thiol ligase activity	2 out of 113 genes, 1.8%	59 out of 25348 genes, 0.2%	1	Bra031265, Bra031263
endoribonuclease activity	2 out of 113 genes, 1.8%	64 out of 25348 genes, 0.3%	1	Bra027179, Bra014136
ligase activity, forming carbon-sulfur bonds	2 out of 113 genes, 1.8%	71 out of 25348 genes, 0.3%	1	Bra031265, Bra031263
galactosyltransferase activity	2 out of 113 genes, 1.8%	75 out of 25348 genes, 0.3%	1	Bra019081, Bra005595
phosphatase activity	6 out of 113 genes, 5.3%	605 out of 25348 genes, 2.4%	1	Bra021870, Bra014764, Bra016025, Bra016206, Bra018167, Bra032335
unfolded protein binding	3 out of 113 genes, 2.7%	196 out of 25348 genes, 0.8%	1	Bra031195, Bra021165, Bra033685
rRNA binding	2 out of 113 genes, 1.8%	88 out of 25348 genes, 0.3%	1	Bra040853, Bra020970

Terms for downstream2k.bed_Down_Gene_P

Gene Ontology term	Cluster frequency	Genome frequency of use	Corrected P-value	Genes annotated to the term
cellular modified amino acid metabolic process	6 out of 125 genes, 4.8%	300 out of 28587 genes, 1.0%	1	Bra032749, Bra031265, Bra031263, Bra019333, Bra035662, Bra033455
primary shoot apical meristem specification	3 out of 125 genes, 2.4%	109 out of 28587 genes, 0.4%	1	Bra021592, Bra014234, Bra014136
positive regulation of translation	2 out of 125 genes, 1.6%	39 out of 28587 genes, 0.1%	1	Bra038106, Bra009325
cellular modified amino acid biosynthetic process	4 out of 125 genes, 3.2%	205 out of 28587 genes, 0.7%	1	Bra031265, Bra031263, Bra019333, Bra035662
embryonic meristem initiation	3 out of 125 genes, 2.4%	120 out of 28587 genes, 0.4%	1	Bra021592, Bra014234, Bra014136
negative regulation of flower development	3 out of 125 genes, 2.4%	128 out of 28587 genes, 0.4%	1	Bra038346, Bra035633, Bra014628
L-serine metabolic process	2 out of 125 genes, 1.6%	48 out of 28587 genes, 0.2%	1	Bra032749, Bra036114
folic acid-containing compound metabolic process	2 out of 125 genes, 1.6%	50 out of 28587 genes, 0.2%	1	Bra032749, Bra035662
sulfur amino acid biosynthetic process	8 out of 125 genes, 6.4%	775 out of 28587 genes,	1	Bra014117, Bra036114, Bra031306, Bra019333, Bra014107, Bra032750, Bra035466, Bra033455

		2.7%		
negative regulation of reproductive process	3 out of 125 genes, 2.4%	134 out of 28587 genes, 0.5%	1	Bra038346, Bra035633, Bra014628

Terms for Intron.bed_Down_Gene_C

Gene Ontology term	Cluster frequency	Genome frequency of use	Corrected P-value	Genes annotated to the term
cell periphery	25 out of 57 genes, 43.9%	8608 out of 33485 genes, 25.7%	0.12768	Bra025640, Bra030913, Bra027548, Bra021173, Bra002392, Bra014082, Bra030915, Bra014404, Bra022247, Bra015873, Bra014431, Bra011094, Bra006799, Bra031296, Bra021217, Bra030250, Bra018183, Bra030455, Bra021868, Bra015685, Bra024598, Bra018663, Bra030692, Bra033685, Bra040998
plant-type cell wall	5 out of 57 genes, 8.8%	742 out of 33485 genes, 2.2%	0.49557	Bra025640, Bra030915, Bra030913, Bra011094, Bra021217
membrane	29 out of 57 genes, 50.9%	11755 out of 33485 genes, 35.1%	0.59980	Bra030913, Bra027548, Bra019504, Bra021173, Bra002392, Bra011900, Bra014082, Bra030915, Bra014404, Bra022247, Bra014431, Bra006799, Bra031296, Bra030250, Bra018183, Bra030455, Bra021868, Bra015685, Bra024598, Bra018663, Bra027046, Bra034939, Bra034156, Bra031306, Bra030692, Bra030328, Bra033685, Bra040998, Bra030896
plant-type vacuole	3 out of 57 genes, 5.3%	264 out of 33485 genes, 0.8%	0.60106	Bra030915, Bra030328, Bra030913
stromule	2 out of 57 genes, 3.5%	115 out of 33485 genes,	0.95709	Bra038030, Bra019504

		0.3%		
cell wall	7 out of 57 genes, 12.3%	1682 out of 33485 genes, 5.0%	1	Bra025640, Bra030915, Bra030913, Bra015873, Bra011094, Bra033685, Bra021217
external encapsulating structure	7 out of 57 genes, 12.3%	1713 out of 33485 genes, 5.1%	1	Bra025640, Bra030915, Bra030913, Bra015873, Bra011094, Bra033685, Bra021217
cytosolic ribosome	4 out of 57 genes, 7.0%	663 out of 33485 genes, 2.0%	1	Bra030915, Bra022247, Bra030913, Bra024598
cytosolic part	4 out of 57 genes, 7.0%	723 out of 33485 genes, 2.2%	1	Bra030915, Bra022247, Bra030913, Bra024598
plasma membrane	19 out of 57 genes, 33.3%	7499 out of 33485 genes, 22.4%	1	Bra027548, Bra021173, Bra002392, Bra014082, Bra014404, Bra022247, Bra014431, Bra006799, Bra031296, Bra030250, Bra018183, Bra030455, Bra021868, Bra015685, Bra024598, Bra018663, Bra030692, Bra033685, Bra040998

Terms for Intron.bed_Down_Gene_F

**Gene Ontology term**	**Cluster frequency**	**Genome frequency of use**	**Corrected P-value**	**Genes annotated to the term**
phosphoric ester hydrolase activity	6 out of 53 genes, 11.3%	772 out of 25348 genes, 3.0%	0.40972	Bra034939, Bra033249, Bra038610, Bra030455, Bra014405, Bra014826
hydrolase activity	19 out of 53 genes, 35.8%	5105 out of 25348 genes,	0.43960	Bra025640, Bra033249, Bra030913, Bra014826, Bra021173, Bra014082, Bra030915, Bra015873, Bra021217, Bra030250, Bra038030, Bra030455,

		20.1%		Bra014405, Bra015685, Bra005795, Bra014108, Bra034939, Bra038610, Bra014620
phosphatase activity	5 out of 53 genes, 9.4%	605 out of 25348 genes, 2.4%	0.65566	Bra033249, Bra038610, Bra030455, Bra014405, Bra014826
hydrolase activity, acting on ester bonds	9 out of 53 genes, 17.0%	1759 out of 25348 genes, 6.9%	0.77711	Bra034939, Bra030915, Bra033249, Bra038610, Bra030913, Bra030455, Bra014405, Bra014826, Bra021217
protein serine/threonine phosphatase activity	3 out of 53 genes, 5.7%	277 out of 25348 genes, 1.1%	1	Bra038610, Bra030455, Bra014405
lipase activity	3 out of 53 genes, 5.7%	283 out of 25348 genes, 1.1%	1	Bra034939, Bra030915, Bra030913
ATP binding	13 out of 53 genes, 24.5%	3422 out of 25348 genes, 13.5%	1	Bra030250, Bra027548, Bra027691, Bra021173, Bra015685, Bra005795, Bra004983, Bra030692, Bra022247, Bra014042, Bra033685, Bra003650, Bra040998
catalytic activity	38 out of 53 genes, 71.7%	14523 out of 25348 genes, 57.3%	1	Bra025640, Bra027548, Bra019504, Bra014826, Bra021173, Bra002392, Bra011900, Bra030915, Bra015873, Bra006799, Bra021217, Bra030250, Bra038030, Bra018183, Bra021868, Bra014405, Bra015685, Bra027046, Bra018663, Bra014108, Bra004856, Bra031306, Bra040998, Bra039755, Bra033249, Bra030913, Bra027691, Bra014082, Bra022247, Bra003650, Bra030455, Bra005795, Bra002967, Bra034939, Bra030692, Bra038610, Bra014620, Bra014042
protein serine/threonine/tyrosine kinase activity	2 out of 53 genes, 3.8%	114 out of 25348 genes, 0.4%	1	Bra018183, Bra027691

adenyl ribonucleotide binding	13 out of 53 genes, 24.5%	3557 out of 25348 genes, 14.0%	1	Bra030250, Bra027548, Bra027691, Bra021173, Bra015685, Bra005795, Bra004983, Bra030692, Bra022247, Bra014042, Bra033685, Bra003650, Bra040998

Terms for Intron.bed_Down_Gene_P

**Gene Ontology term**	**Cluster frequency**	**Genome frequency of use**	**Corrected P-value**	**Genes annotated to the term**
S-glycoside catabolic process	3 out of 56 genes, 5.4%	70 out of 28587 genes, 0.2%	0.19283	Bra030915, Bra030913, Bra021173
glycosinolate catabolic process	3 out of 56 genes, 5.4%	70 out of 28587 genes, 0.2%	0.19283	Bra030915, Bra030913, Bra021173
glucosinolate catabolic process	3 out of 56 genes, 5.4%	70 out of 28587 genes, 0.2%	0.19283	Bra030915, Bra030913, Bra021173
phosphate-containing compound metabolic process	21 out of 56 genes, 37.5%	5265 out of 28587 genes, 18.4%	0.33047	Bra039755, Bra033249, Bra030913, Bra027548, Bra014826, Bra027691, Bra021173, Bra030915, Bra003650, Bra038030, Bra018183, Bra030455, Bra014405, Bra005795, Bra024598, Bra027046, Bra002967, Bra034939, Bra030692, Bra014042, Bra040998
sulfur compound catabolic process	3 out of 56 genes, 5.4%	91 out of 28587 genes, 0.3%	0.41569	Bra030915, Bra030913, Bra021173
cellular glucan metabolic process	8 out of 56 genes, 14.3%	1034 out of 28587 genes, 3.6%	0.47135	Bra039755, Bra030913, Bra021173, Bra011900, Bra024598, Bra014108, Bra030915, Bra030328

glucan metabolic process	8 out of 56 genes, 14.3%	1034 out of 28587 genes, 3.6%	0.47135	Bra039755, Bra030913, Bra021173, Bra011900, Bra024598, Bra014108, Bra030915, Bra030328
phosphorus metabolic process	21 out of 56 genes, 37.5%	5430 out of 28587 genes, 19.0%	0.50309	Bra039755, Bra033249, Bra030913, Bra027548, Bra014826, Bra027691, Bra021173, Bra030915, Bra003650, Bra038030, Bra018183, Bra030455, Bra014405, Bra005795, Bra024598, Bra027046, Bra002967, Bra034939, Bra030692, Bra014042, Bra040998
cellular carbohydrate metabolic process	10 out of 56 genes, 17.9%	1733 out of 28587 genes, 6.1%	0.97564	Bra039755, Bra030913, Bra014826, Bra021173, Bra011900, Bra024598, Bra014108, Bra030692, Bra030915, Bra030328
polysaccharide metabolic process	10 out of 56 genes, 17.9%	1787 out of 28587 genes, 6.3%	1	Bra039755, Bra033249, Bra030913, Bra021173, Bra011900, Bra024598, Bra014108, Bra030692, Bra030915, Bra030328

Terms for upstream2k.bed_Down_Gene_C

**Gene Ontology term**	**Cluster frequency**	**Genome frequency of use**	**Corrected P-value**	**Genes annotated to the term**
tubulin complex	3 out of 219 genes, 1.4%	26 out of 33485 genes, 0.1%	0.06997	Bra018184, Bra014232, Bra020062
vacuolar membrane	20 out of 219 genes, 9.1%	1394 out of 33485 genes, 4.2%	0.09715	Bra014626, Bra018719, Bra030913, Bra019504, Bra022757, Bra038669, Bra030911, Bra035433, Bra015836, Bra014232, Bra021542, Bra025210, Bra005522, Bra035847, Bra016295, Bra019790, Bra030921, Bra039708, Bra021517, Bra015839
vacuolar part	20 out of 219 genes, 9.1%	1406 out of 33485 genes,	0.10776	Bra014626, Bra018719, Bra030913, Bra019504, Bra022757, Bra038669, Bra030911, Bra035433, Bra015836, Bra014232, Bra021542, Bra025210,

		4.2%		Bra005522, Bra035847, Bra016295, Bra019790, Bra030921, Bra039708, Bra021517, Bra015839
non-membrane-bounded organelle	26 out of 219 genes, 11.9%	2254 out of 33485 genes, 6.7%	0.38692	Bra003776, Bra030913, Bra022757, Bra028373, Bra030911, Bra010455, Bra039374, Bra014096, Bra021593, Bra014232, Bra003871, Bra020062, Bra021542, Bra005522, Bra031983, Bra014763, Bra033820, Bra005395, Bra016295, Bra024598, Bra010456, Bra018184, Bra030921, Bra018145, Bra040213, Bra021517
intracellular non-membrane-bounded organelle	26 out of 219 genes, 11.9%	2254 out of 33485 genes, 6.7%	0.38692	Bra003776, Bra030913, Bra022757, Bra028373, Bra030911, Bra010455, Bra039374, Bra014096, Bra021593, Bra014232, Bra003871, Bra020062, Bra021542, Bra005522, Bra031983, Bra014763, Bra033820, Bra005395, Bra016295, Bra024598, Bra010456, Bra018184, Bra030921, Bra018145, Bra040213, Bra021517
plant-type vacuole	6 out of 219 genes, 2.7%	264 out of 33485 genes, 0.8%	0.87866	Bra027030, Bra030913, Bra021542, Bra025210, Bra030911, Bra015839
organelle membrane	25 out of 219 genes, 11.4%	2429 out of 33485 genes, 7.3%	1	Bra014626, Bra018719, Bra032697, Bra038029, Bra030913, Bra019504, Bra022757, Bra038669, Bra030911, Bra035433, Bra033473, Bra015836, Bra014232, Bra021542, Bra005522, Bra025210, Bra014210, Bra035847, Bra016295, Bra019790, Bra030921, Bra039708, Bra017476, Bra021517, Bra015839
chloroplast photosystem II	2 out of 219 genes, 0.9%	32 out of 33485 genes, 0.1%	1	Bra039720, Bra030537
membrane	92 out of 219 genes, 42.0%	11755 out of 33485 genes, 35.1%	1	Bra014626, Bra033790, Bra014087, Bra027548, Bra032034, Bra030911, Bra033473, Bra015920, Bra018001, Bra005446, Bra015984, Bra018183, Bra033983, Bra035458, Bra019790, Bra024598, Bra027178, Bra039720, Bra026922, Bra005592, Bra017476, Bra030896, Bra018719, Bra014233,

				Bra032711, Bra030913, Bra038669, Bra014082, Bra035433, Bra017563, Bra012786, Bra010455, Bra033893, Bra004857, Bra033865, Bra012359, Bra005522, Bra025210, Bra014763, Bra005444, Bra038223, Bra005395, Bra016207, Bra016295, Bra033245, Bra040213, Bra014136, Bra030537, Bra003776, Bra032697, Bra038029, Bra019504, Bra021490, Bra032696, Bra021593, Bra035847, Bra032695, Bra013038, Bra019634, Bra040406, Bra014110, Bra021367, Bra036730, Bra039708, Bra039662, Bra034880, Bra003923, Bra014122, Bra040998, Bra015839, Bra030919, Bra005441, Bra022757, Bra015817, Bra009509, Bra015836, Bra018309, Bra014232, Bra020062, Bra014210, Bra021542, Bra034289, Bra036323, Bra034291, Bra032749, Bra016143, Bra022104, Bra031011, Bra013747, Bra030921, Bra014259, Bra021517
intracellular organelle part	55 out of 219 genes, 25.1%	6486 out of 33485 genes, 19.4%	1	Bra003776, Bra014626, Bra032697, Bra038029, Bra019504, Bra028373, Bra030911, Bra033473, Bra021593, Bra035466, Bra035847, Bra038030, Bra030251, Bra013038, Bra032695, Bra040406, Bra019790, Bra024598, Bra039720, Bra039708, Bra017476, Bra003923, Bra015839, Bra031242, Bra014233, Bra018719, Bra030913, Bra022757, Bra028339, Bra038669, Bra030925, Bra035433, Bra010455, Bra015836, Bra014096, Bra014232, Bra020062, Bra021542, Bra025210, Bra014210, Bra005522, Bra014763, Bra033820, Bra005395, Bra026691, Bra016295, Bra013747, Bra010456, Bra018184, Bra030921, Bra018145, Bra040213, Bra021517, Bra014803, Bra030537

Terms for upstream2k.bed_Down_Gene_F

Gene Ontology term	Cluster frequency	Genome frequency of use	Corrected P-value	Genes annotated to the term
GTPase activity	9 out of 164	237 out of	0.00414	Bra005395, Bra018147, Bra034879, Bra035433, Bra018184, Bra033865,

	genes, 5.5%	25348 genes, 0.9%		Bra014232, Bra004857, Bra020062
L-malate dehydrogenase activity	3 out of 164 genes, 1.8%	13 out of 25348 genes, 0.1%	0.01189	Bra039662, Bra019504, Bra019790
malate dehydrogenase activity	3 out of 164 genes, 1.8%	24 out of 25348 genes, 0.1%	0.07987	Bra039662, Bra019504, Bra019790
structural constituent of cytoskeleton	4 out of 164 genes, 2.4%	63 out of 25348 genes, 0.2%	0.12260	Bra018184, Bra005395, Bra014232, Bra020062
GTP binding	9 out of 164 genes, 5.5%	440 out of 25348 genes, 1.7%	0.38474	Bra005395, Bra018147, Bra034879, Bra035433, Bra018184, Bra033865, Bra014232, Bra004857, Bra020062
guanyl ribonucleotide binding	9 out of 164 genes, 5.5%	440 out of 25348 genes, 1.7%	0.38474	Bra005395, Bra018147, Bra034879, Bra035433, Bra018184, Bra033865, Bra014232, Bra004857, Bra020062
guanyl nucleotide binding	9 out of 164 genes, 5.5%	442 out of 25348 genes, 1.7%	0.39658	Bra005395, Bra018147, Bra034879, Bra035433, Bra018184, Bra033865, Bra014232, Bra004857, Bra020062
thalian-diol desaturase activity	2 out of 164 genes, 1.2%	16 out of 25348 genes, 0.1%	0.77145	Bra038254, Bra014143
catalytic activity	110 out of 164 genes, 67.1%	14523 out of 25348 genes, 57.3%	1	Bra035436, Bra033790, Bra033453, Bra027548, Bra028373, Bra034879, Bra030911, Bra005879, Bra039374, Bra015920, Bra015873, Bra018001, Bra005446, Bra035466, Bra038030, Bra005596, Bra040633, Bra015984, Bra018183, Bra003849, Bra027030, Bra033983, Bra030251, Bra021164, Bra019790, Bra039720, Bra038254, Bra005592, Bra021218, Bra032748, Bra031010, Bra032711, Bra030913, Bra019319, Bra027271, Bra014082, Bra035433, Bra017563, Bra010455, Bra032731, Bra033893, Bra004857, Bra033865, Bra005522, Bra023907, Bra040405, Bra005395, Bra016207,

				Bra038093, Bra022704, Bra031266, Bra033245, Bra040213, Bra038222, Bra003945, Bra014136, Bra032697, Bra014839, Bra019504, Bra021490, Bra020982, Bra040862, Bra032696, Bra031263, Bra026271, Bra032674, Bra035847, Bra019817, Bra019634, Bra040406, Bra014405, Bra003775, Bra014110, Bra038102, Bra039486, Bra039708, Bra039662, Bra004841, Bra028803, Bra014122, Bra040998, Bra014080, Bra005158, Bra022757, Bra017675, Bra005283, Bra038361, Bra030925, Bra021740, Bra009509, Bra014144, Bra014232, Bra020062, Bra021542, Bra014210, Bra034289, Bra032749, Bra033820, Bra016143, Bra018175, Bra018147, Bra013747, Bra002967, Bra018184, Bra030921, Bra023883, Bra014042, Bra014143, Bra021517, Bra014803
G-protein coupled receptor activity	2 out of 164 genes, 1.2%	19 out of 25348 genes, 0.1%	1	Bra018719, Bra015817

Terms for upstream2k.bed_Down_Gene_P

**Gene Ontology term**	**Cluster frequency**	**Genome frequency of use**	**Corrected P-value**	**Genes annotated to the term**
carbohydrate derivative catabolic process	14 out of 198 genes, 7.1%	579 out of 28587 genes, 2.0%	0.05645	Bra005395, Bra030913, Bra028373, Bra021164, Bra034879, Bra030911, Bra005283, Bra035433, Bra018184, Bra004857, Bra033865, Bra014232, Bra020062, Bra021542
GTP catabolic process	8 out of 198 genes, 4.0%	191 out of 28587 genes, 0.7%	0.05896	Bra005395, Bra034879, Bra035433, Bra018184, Bra033865, Bra014232, Bra004857, Bra020062
guanosine-containing compound catabolic process	8 out of 198 genes, 4.0%	191 out of 28587 genes, 0.7%	0.05896	Bra005395, Bra034879, Bra035433, Bra018184, Bra033865, Bra014232, Bra004857, Bra020062

GTP metabolic process	8 out of 198 genes, 4.0%	201 out of 28587 genes, 0.7%	0.08425	Bra005395, Bra034879, Bra035433, Bra018184, Bra033865, Bra014232, Bra004857, Bra020062
glycosyl compound catabolic process	13 out of 198 genes, 6.6%	536 out of 28587 genes, 1.9%	0.10109	Bra005395, Bra030913, Bra028373, Bra034879, Bra030911, Bra005283, Bra035433, Bra018184, Bra004857, Bra033865, Bra014232, Bra020062, Bra021542
guanosine-containing compound metabolic process	8 out of 198 genes, 4.0%	224 out of 28587 genes, 0.8%	0.17775	Bra005395, Bra034879, Bra035433, Bra018184, Bra033865, Bra014232, Bra004857, Bra020062
maintenance of seed dormancy	2 out of 198 genes, 1.0%	6 out of 28587 genes, 0.0%	0.70158	Bra018719, Bra038102
maintenance of dormancy	2 out of 198 genes, 1.0%	6 out of 28587 genes, 0.0%	0.70158	Bra018719, Bra038102
primary metabolic process	133 out of 198 genes, 67.2%	16037 out of 28587 genes, 56.1%	0.91659	Bra033453, Bra027548, Bra011515, Bra034879, Bra033473, Bra005879, Bra039374, Bra018001, Bra003871, Bra035466, Bra031983, Bra015984, Bra030251, Bra021215, Bra021164, Bra024598, Bra038254, Bra005592, Bra017476, Bra018719, Bra031010, Bra032711, Bra030913, Bra019319, Bra035433, Bra017563, Bra010455, Bra014096, Bra033865, Bra005522, Bra021108, Bra014763, Bra005395, Bra026691, Bra016207, Bra022557, Bra031266, Bra033245, Bra040213, Bra038222, Bra030537, Bra014136, Bra032697, Bra014839, Bra038029, Bra021490, Bra031263, Bra031985, Bra026885, Bra013038, Bra014405, Bra021367, Bra014110, Bra038102, Bra028803, Bra014122, Bra022757, Bra005441, Bra017675, Bra005283, Bra038361, Bra030925, Bra018309, Bra013997, Bra014232, Bra021542, Bra033454, Bra002967, Bra014042, Bra038696, Bra018145, Bra014143, Bra035436, Bra028373, Bra030911, Bra015920, Bra005446, Bra005596, Bra038030, Bra018183, Bra033983, Bra027030, Bra003699, Bra021218, Bra032748, Bra032768, Bra027271, Bra004857, Bra033893, Bra025210,

				Bra028114, Bra023907, Bra033866, Bra039551, Bra016295, Bra003776, Bra020982, Bra019504, Bra021593, Bra032674, Bra035847, Bra003775, Bra033468, Bra040406, Bra019634, Bra039486, Bra039708, Bra039662, Bra033458, Bra040998, Bra015839, Bra030249, Bra030919, Bra010083, Bra036757, Bra028339, Bra031269, Bra032170, Bra014144, Bra035594, Bra014210, Bra020062, Bra034289, Bra032749, Bra033820, Bra016143, Bra018175, Bra018147, Bra013747, Bra018184, Bra030921, Bra014803, Bra021517
tricyclic triterpenoid metabolic process	3 out of 198 genes, 1.5%	28 out of 28587 genes, 0.1%	0.94171	Bra038254, Bra014144, Bra014143

Supplementary Table S6. Top 10 significant KEGG pathways involving up-methylated differentially methylated genes in CA plants.

CDS.bed_Up_Gene

#	Pathway	DEGs with pathway annotation	All genes with pathway annotation	Differentially expressed genes	Qvalue	Pathway ID
1	Homologous recombination	3 (5.45%)	102 (0.51%)	Bra003643, Bra032088, Bra035691	0.1075179	ko03440
2	Biotin metabolism	1 (1.82%)	6 (0.03%)	Bra004758	0.2280586	ko00780
3	Mismatch repair	2 (3.64%)	74 (0.37%)	Bra003643, Bra032088	0.2280586	ko03430
4	DNA replication	2 (3.64%)	93 (0.46%)	Bra003643, Bra032088	0.2346595	ko03030
5	Plant-pathogen interaction	8 (14.55%)	1402 (6.98%)	Bra003778, Bra003858, Bra018609, Bra020298, Bra021579, Bra021754, Bra028581, Bra040061	0.2346595	ko04626
6	Nucleotide excision repair	2 (3.64%)	116 (0.58%)	Bra003643, Bra032088	0.2346595	ko03420
7	Zeatin biosynthesis	2 (3.64%)	126 (0.63%)	Bra031343, Bra036804	0.2346595	ko00908
8	Sulfur relay system	1 (1.82%)	21 (0.1%)	Bra030447	0.2346595	ko04122
9	Folate biosynthesis	1 (1.82%)	21 (0.1%)	Bra035662	0.2346595	ko00790
10	Plant hormone signal transduction	7 (12.73%)	1285 (6.4%)	Bra003778, Bra021579, Bra027169, Bra029934, Bra030930, Bra039364, Bra040061	0.2346595	ko04075

downstream2k.bed_Up_Gene

#	Pathway	DEGs with pathway annotation	All genes with pathway annotation	Differentially expressed genes	Qvalue	Pathway ID
1	Protein export	5 (3.97%)	108 (0.54%)	Bra014039, Bra014189, Bra022517, Bra035669, Bra037545	0.0334807 3	ko03060
2	ABC transporters	5 (3.97%)	234 (1.17%)	Bra010773, Bra018132, Bra020299, Bra021173,	0.4514837	ko02010

				Bra035394	6	
3	Fructose and mannose metabolism	3 (2.38%)	106 (0.53%)	Bra016184, Bra016799, Bra038519	0.5307064 8	ko00051
4	Zeatin biosynthesis	3 (2.38%)	126 (0.63%)	Bra011492, Bra011495, Bra031343	0.5307064 8	ko00908
5	Plant-pathogen interaction	14 (11.11%)	1402 (6.98%)	Bra003858, Bra005168, Bra014188, Bra020724, Bra028581, Bra032741, Bra032746, Bra033474, Bra033476, Bra034257, Bra035366, Bra037254, Bra037470, Bra038935	0.5307064 8	ko04626
6	Plant hormone signal transduction	13 (10.32%)	1285 (6.4%)	Bra003665, Bra005168, Bra008806, Bra016069, Bra019524, Bra020299, Bra020994, Bra034257, Bra035366, Bra038935, Bra039364, Bra039732, Bra039855	0.5307064 8	ko04075
7	Fatty acid biosynthesis	2 (1.59%)	67 (0.33%)	Bra003777, Bra039178	0.5307064 8	ko00061
8	Mismatch repair	2 (1.59%)	74 (0.37%)	Bra031722, Bra032088	0.5516698 6	ko03430
9	Nitrogen metabolism	2 (1.59%)	83 (0.41%)	Bra028132, Bra035392	0.5961409 7	ko00910
10	DNA replication	2 (1.59%)	93 (0.46%)	Bra031722, Bra032088	0.6279630 0	ko03030

Intron.bed_Up_Gene

#	Pathway	DEGs with pathway annotation	All genes with pathway annotation	Differentially expressed genes	Qvalue	Pathway ID
1	Homologous recombination	3 (7.14%)	102 (0.51%)	Bra003643, Bra032088, Bra035691	0.04681628	ko03440

2	Sesquiterpenoid and triterpenoid biosynthesis	1 (2.38%)	4 (0.02%)	Bra040920	0.11990183	ko00909
3	Mismatch repair	2 (4.76%)	74 (0.37%)	Bra003643, Bra032088	0.11990183	ko03430
4	Flavonoid biosynthesis	3 (7.14%)	241 (1.2%)	Bra019636, Bra021492, Bra032743	0.11990183	ko00941
5	DNA replication	2 (4.76%)	93 (0.46%)	Bra003643, Bra032088	0.11990183	ko03030
6	Nucleotide excision repair	2 (4.76%)	116 (0.58%)	Bra003643, Bra032088	0.15115869	ko03420
7	Sulfur relay system	1 (2.38%)	21 (0.1%)	Bra030447	0.19906333	ko04122
8	Folate biosynthesis	1 (2.38%)	21 (0.1%)	Bra035662	0.19906333	ko00790
9	Plant hormone signal transduction	6 (14.29%)	1285 (6.4%)	Bra003778, Bra018213, Bra021579, Bra027169, Bra029934, Bra030930	0.20358370	ko04075
10	Selenocompound metabolism	1 (2.38%)	36 (0.18%)	Bra040811	0.26888407	ko00450

upstream2k.bed_Up_Gene

#	Pathway	DEGs with pathway annotation	All genes with pathway annotation	Differentially expressed genes	Qvalue	Pathway ID
1	Sulfur relay system	2 (1.28%)	21 (0.1%)	Bra028125, Bra030447	0.6817097	ko04122
2	Cyanoamino acid metabolism	4 (2.56%)	131 (0.65%)	Bra003928, Bra020551, Bra028383, Bra035508	0.6817097	ko00460
3	Biotin metabolism	1 (0.64%)	6 (0.03%)	Bra004758	0.7438822	ko00780
4	Tryptophan metabolism	4 (2.56%)	179 (0.89%)	Bra017364, Bra019522, Bra019538, Bra035508	0.7438822	ko00380
5	Other glycan degradation	3 (1.92%)	119 (0.59%)	Bra020216, Bra024237, Bra030917	0.7438822	ko00511
6	Regulation of autophagy	3 (1.92%)	132 (0.66%)	Bra014787, Bra025605, Bra031233	0.7438822	ko04140
7	Protein processing in endoplasmic reticulum	6 (3.85%)	467 (2.33%)	Bra010967, Bra014319, Bra020964, Bra021213, Bra030275, Bra032766	0.7438822	ko04141

8	mRNA surveillance pathway	4 (2.56%)	280 (1.39%)	Bra005496, Bra030275, Bra031294, Bra040136	0.7438822	ko03015
9	Galactose metabolism	2 (1.28%)	102 (0.51%)	Bra014254, Bra024237	0.7438822	ko00052
10	Plant hormone signal transduction	13 (8.33%)	1285 (6.4%)	Bra003778, Bra003973, Bra004329, Bra005525, Bra005529, Bra014787, Bra019558, Bra019768, Bra021245, Bra021579, Bra029934, Bra030930, Bra035381	0.7438822	ko04075

Supplementary Table S7. Top 10 significant KEGG pathways involving down-methylated differentially methylated genes in CA plants.

CDS.bed_Down_Gene

#	Pathway	DEGs with pathway annotation	All genes with pathway annotation	Differentially expressed genes	Qvalue	Pathway ID
1	Plant-pathogen interaction	8 (14.55%)	1402 (6.98%)	Bra005534, Bra005563, Bra013999, Bra021868, Bra021913, Bra032746, Bra038596, Bra040998	0.5131311	ko04626
2	Phosphatidylinositol signaling system	2 (3.64%)	147 (0.73%)	Bra027046, Bra027271	0.5131311	ko04070
3	Circadian rhythm - plant	2 (3.64%)	153 (0.76%)	Bra004856, Bra027548	0.5131311	ko04712
4	Purine metabolism	3 (5.45%)	342 (1.7%)	Bra021585, Bra032731, Bra038030	0.5131311	ko00230
5	Pentose and glucuronate interconversions	3 (5.45%)	354 (1.76%)	Bra014620, Bra021217, Bra030511	0.5131311	ko00040
6	Starch and sucrose metabolism	4 (7.27%)	594 (2.96%)	Bra011900, Bra014620, Bra021217, Bra030511	0.5131311	ko00500
7	One carbon pool by folate	1 (1.82%)	36 (0.18%)	Bra038030	0.5131311	ko00670
8	Glycerophospholipid metabolism	3 (5.45%)	449 (2.24%)	Bra027046, Bra033249, Bra034939	0.5131311	ko00564
9	Sulfur metabolism	1 (1.82%)	56 (0.28%)	Bra005879	0.5131311	ko00920
10	Ubiquinone and other terpenoid-quinone biosynthesis	1 (1.82%)	63 (0.31%)	Bra031263	0.5131311	ko00130

downstream2k.bed_Down_Gene

#	Pathway	DEGs with pathway annotation (90)	All genes with pathway annotation (20079)	Differentially expressed genes	Qvalue	Pathway ID
1	Carotenoid biosynthesis	3 (3.33%)	138 (0.69%)	Bra020970, Bra030912, Bra032770	0.6752230	ko00906

2	Ubiquinone and other terpenoid-quinone biosynthesis	2 (2.22%)	63 (0.31%)	Bra031263, Bra031265	0.6752230	ko00130
3	Folate biosynthesis	1 (1.11%)	21 (0.1%)	Bra035662	0.6752230	ko00790
4	Vitamin B6 metabolism	1 (1.11%)	22 (0.11%)	Bra016025	0.6752230	ko00750
5	Linoleic acid metabolism	1 (1.11%)	28 (0.14%)	Bra013690	0.6752230	ko00591
6	Photosynthesis - antenna proteins	1 (1.11%)	34 (0.17%)	Bra014433	0.6752230	ko00196
7	RNA polymerase	2 (2.22%)	151 (0.75%)	Bra021519, Bra021585	0.6752230	ko03020
8	One carbon pool by folate	1 (1.11%)	36 (0.18%)	Bra032749	0.6752230	ko00670
9	Cysteine and methionine metabolism	2 (2.22%)	159 (0.79%)	Bra032750, Bra036114	0.6752230	ko00270
10	Histidine metabolism	1 (1.11%)	40 (0.2%)	Bra035466	0.6752230	ko00340

Intron.bed_Down_Gene

#	Pathway	DEGs with pathway annotation (44)	All genes with pathway annotation (20079)	Differentially expressed genes	Qvalue	Pathway ID
1	Circadian rhythm - plant	2 (4.55%)	153 (0.76%)	Bra004856, Bra027548	0.4802441	ko04712
2	Glycerophospholipid metabolism	3 (6.82%)	449 (2.24%)	Bra027046, Bra033249, Bra034939	0.4802441	ko00564
3	One carbon pool by folate	1 (2.27%)	36 (0.18%)	Bra038030	0.4802441	ko00670
4	Tropane, piperidine and pyridine alkaloid biosynthesis	1 (2.27%)	57 (0.28%)	Bra039755	0.4802441	ko00960
5	Ribosome biogenesis in eukaryotes	2 (4.55%)	282 (1.4%)	Bra004856, Bra027548	0.4802441	ko03008
6	Glyoxylate and dicarboxylate metabolism	1 (2.27%)	66 (0.33%)	Bra019504	0.4802441	ko00630

7	Starch and sucrose metabolism	3 (6.82%)	594 (2.96%)	Bra011900, Bra014620, Bra021217	0.4802441	ko00500
8	Nitrogen metabolism	1 (2.27%)	83 (0.41%)	Bra022247	0.4802441	ko00910
9	Pentose and glucuronate interconversions	2 (4.55%)	354 (1.76%)	Bra014620, Bra021217	0.4802441	ko00040
10	Glycerolipid metabolism	1 (2.27%)	104 (0.52%)	Bra033249	0.4802441	ko00561

upstream2k.bed_Down_Gene

#	Pathway	DEGs with pathway annotation (135)	All genes with pathway annotation (20079)	Differentially expressed genes	Qvalue	Pathway ID
1	Phagosome	8 (5.93%)	232 (1.16%)	Bra004857, Bra005395, Bra005441, Bra014232, Bra018184, Bra020062, Bra027271, Bra038669	0.0118651 9	ko04145
2	Citrate cycle (TCA cycle)	5 (3.7%)	112 (0.56%)	Bra014144, Bra019504, Bra019790, Bra022757, Bra039662	0.0322422 5	ko00020
3	Carbon fixation in photosynthetic organisms	5 (3.7%)	133 (0.66%)	Bra019504, Bra019634, Bra019790, Bra039662, Bra040406	0.0345281 0	ko00710
4	Biosynthesis of secondary metabolites	26 (19.26%)	2155 (10.73%)	Bra005158, Bra005522, Bra005596, Bra005879, Bra014143, Bra014144, Bra017675, Bra018147, Bra019504, Bra019634, Bra019790, Bra021490, Bra022757, Bra030911, Bra031263, Bra031266, Bra032697, Bra032749, Bra035466, Bra035847, Bra038030, Bra038102, Bra038254, Bra039662, Bra040213, Bra040406	0.0345281 0	ko01110
5	Pyruvate metabolism	5 (3.7%)	140 (0.7%)	Bra019504, Bra019634, Bra019790, Bra022757, Bra039662	0.0345281 0	ko00620
6	Glyoxylate and dicarboxylate metabolism	3 (2.22%)	66 (0.33%)	Bra019504, Bra019790, Bra039662	0.1115682 4	ko00630

7	Regulation of autophagy	4 (2.96%)	132 (0.66%)	Bra005446, Bra014122, Bra033820, Bra033893	0.11660594	ko04140
8	One carbon pool by folate	2 (1.48%)	36 (0.18%)	Bra032749, Bra038030	0.20394406	ko00670
9	Purine metabolism	6 (4.44%)	342 (1.7%)	Bra017675, Bra019634, Bra030925, Bra032731, Bra035847, Bra038030	0.21013701	ko00230
10	Metabolic pathways	39 (28.89%)	4416 (21.99%)	Bra003699, Bra003775, Bra003945, Bra005158, Bra005522, Bra005596, Bra014000, Bra014143, Bra014144, Bra014810, Bra017675, Bra018147, Bra018175, Bra019504, Bra019634, Bra019790, Bra021490, Bra022704, Bra022757, Bra023907, Bra027030, Bra030537, Bra030911, Bra030925, Bra031252, Bra031263, Bra031266, Bra032697, Bra032731, Bra032749, Bra035466, Bra035847, Bra038030, Bra038102, Bra038254, Bra039662, Bra039708, Bra040213, Bra040406	0.24191884	ko01100
